# Movement traits important to conservation and fisheries management: an example with red snapper

**DOI:** 10.1038/s41598-025-86892-1

**Published:** 2025-02-07

**Authors:** Susan K. Lowerre-Barbieri, Kara Wall, Claudia Friess, Sean Keenan, Chad Lembke, Joseph Tarnecki, Laura Jay Williams-Grove, William F. Patterson

**Affiliations:** 1 Fisheries and Aquatic Sciences, University of Florida, 7922 NW 71st Street, Gainesville, FL 32653 USA; 2 Florida Fish and Wildlife Research Institute, 100 8th Avenue SE, St. Petersburg, FL 33701 USA; 3 College of Marine Science, University of South Florida, 830 1st St. S, St. Petersburg, FL 33701 USA; 4 Southeast Fisheries Science Center, NOAA Fisheries, 75 Virginia Beach Dr, Miami, FL 33149 USA

**Keywords:** Ecology, Ocean sciences

## Abstract

Site fidelity, space use, and dispersal are commonly estimated with acoustic telemetry (AT) to help inform management and conservation. These behaviors can change with age, habitat and environmental conditions and our ability to accurately estimate them is affected by a study’s *inference power* (design components that affect how accurately detection data represents a species’ movements). Red snapper (*Lutjanus campechanus*) have been extensively studied with AT over a range of time periods and regions, although primarily at artificial reefs (AR). Here, we use large (> 12 km^2^) acoustic positioning arrays to monitor a study area with low-relief hard bottom, a reef ledge, and an AR. Annual fidelity to the study area was estimated to be 54%, but estimates were affected by fate uncertainty and model choice. Emigration increased with storms and in early summer. Abundance was greatest at small habitat patches but space use did not scale with patch size. Although uncommon, long-distance movements and connectivity between habitats occurred, with a maximum dispersal of 206 km. Previous red snapper AT studies varied greatly in array size, study duration, and number of fish tracked, impacting inference power. This made it difficult to compare results and highlights the need for greater standardization in AT methods.

**Supplementary Information:**

The online version contains supplementary material available at 10.1038/s41598-025-86892-1.

## Introduction

Species exhibit eco-evolutionary movement strategies made up of multiple traits occurring at different temporal scales and cued by proximate and ultimate drivers. Movement traits important to conservation and fisheries management are closely tied to habitat selection, which often differs with life stage, food availability, reproduction, and environmental stressors^1,2^. Animals that use different habitats with life stage are predicted to undergo ontogenetic habitat shifts as predation risk or foraging abilities change with size or age^3^, or in many fish, when they reach sexual maturity^4^. Adult movement types range from highly resident to highly migratory or nomadic and are driven by whether adults select the same or different habitat for feeding, breeding, and overwintering^1^. Species which have specific habitat needs associated with adult survival and reproductive success often aggregate in large numbers. For marine fish this increases vulnerability to fishing gear and anthropogenic perturbations, such as oil spills, construction, and mining.

The most common method to track marine fish is acoustic telemetry^5^ (AT) and multiple review articles highlight the importance of AT movement data to help inform management and conservation efforts^6–9^. AT uses electronic tags, which transmit an acoustic signal which is detected by logging stations (i.e., receivers), when the tag is within range. Multiple receivers are typically deployed in an array. AT results are driven by the probability of detection, which differs with many things including movement rate, array design, and background noise (biological, natural—such as sounds associated with weather and currents, and industrial). Array designs fall along a continuum in terms of receiver density (affecting location accuracy), and area monitored. At one end of the continuum are arrays with multiple receivers deployed over large areas which monitor presence/absence at specific sites. At the other end of the continuum are high-density acoustic positioning system (APS) arrays designed for positioning based on triangulation (i.e., signals detected on ≥ three receivers). Triangulation provides high location accuracy, often within a few meters, but the receiver density needed typically results in monitoring only a small area^10^ (≤ 2 km^2^). Most arrays fall somewhere in the middle of these two extremes, and centers of activity (COAs)^11^ are the most common estimate used to assign fish location. This method utilizes time bins of sufficient duration to allow signals to be detected on multiple receivers. Locations are estimated based on the average of the receiver locations that detected the tag within each time bin^12^. We refer to “positions” when triangulation is used and “location” for other, less precise estimates.

Management measures that need movement data include protected areas (both long-term and seasonal), evaluating the impact of introduced habitat such as artificial reefs (AR) or wind farms^13–15^, and protecting against habitat fragmentation^16^. To effectively apply these strategies, there is a growing need to identify quantifiable movement traits and their causes, and to report these results using standards and consistent terminology (Table 1) to make it easier to interpret results. Three key metrics are commonly used to assess movement across different spatio-temporal scales: site fidelity, space use, and dispersal. Because these traits have been extensively studied with AT in red snapper (*Lutjanus campechanus*)^8,17^ (Table 2), we use this species as a case study to evaluate movement and management.

**Table 1:** Definitions of common terms used with processes discussed in this paper.

Term	Definition	References
Habitat	The resources and conditions present in an area that produce occupancy often associated with bottom habitat in marine fish.	Krausman and Morrison 2016^85^
Eco-evolutionary movement strategy	The measurable movement attributes and traits, and the ultimate and proximate cues that drive them, for a given species or population.	Riotte-Lampert & Matthiopoulos, 2020^2^
Life cycle space use	The habitats used throughout a species’ life cycle, which may differ with life stage, and with adult foraging, overwintering, and breeding. The degree of habitat overlap and/or distance between disparate habitats is species-specific.	This study
Fate assignment	The use of movement patterns and recapture data to assign each tagged fish as either dead (predation, capture removal, or unknown cause) or alive (in the study area or emigrated).	Villegas-Rios et al. 2020^35^
Inference power	The accuracy with which detection data reflects a species’ movements due to array size, density, and duration of deployment; number and size of fish tracked; overlap between area monitored and daily space use; and the ability to correctly assign fate.	This study
Adult movement type	The degree and consistency of space used at the annual scale, ranging from highly resident to highly migratory or nomadic.	Lowerre-Barbieri et al. 2021^1^
Movement metric	Site fidelity (or residence), space use, and dispersal	Mueller & Fagan 2008^86^; Allen and Singh 2016^13^
Ontogenetic habitat shift	Shifts in habitat selection over the course of the life cycle due to changes in foraging abilities, reproductive state, and/or vulnerability to predation associated with size or age.	Werner and Gilliam 1984^3^; Fokkema et al., 2020^87^; Lowerre-Barbieri et al.^1^
Home range	The area traversed by the individual in food gathering, mating, and caring for the young.	Burt 1943^88^
Site fidelity	Measures how long an individual remains within a defined space for a predefined time period, typically a year, with residency typically defined as how long it takes for 50% of tagged fish to leave the predefined area.	Grove-Williams & Szedlmayer 2020^17^
Space use	The proportion of detections that fall within a given level or isopleth, typically the 50% area and 95% area and the time period associated with these, typically daily or study duration.	Kraft et al. 2023^49^, this study
Dispersal	Refers to any movement of individuals from a source location (e.g. birth or breeding site) to another site where establishment and reproduction may occur	Clobert et al., 2012^64^; Manel et al., 2023^89^

**Table 2:** Previous studies on red snapper movements with a measure of site fidelity.

Tags deployed (tracked)	Mean TL (range)	APS	Total # *R*	Array DD	Max-imum depth	Estimated detection probabilities	Site fidelity	Fates assigned
102 (85)^33^	639 mm (501–860)	6 A (5R)	30	1,639	30 m	65% at 700 m	Annual: 72%	C, D, E, A, U
46 (17)^34^	578 mm (508–719)	3 A (5R)	15	695	30 m	100% at 400 m	Annual: 82%	C, D, E, A, U
82 (56)^61^	605 mm (454–877)	3 A (5R)	38	1,097	35 m	65% at 700 m	88% @ 10 mths	Tracked, lost, stationary
11 (7)^59^	595 mm (489–702)	1 A (5R)	30	745	28 m	65% at 700 m	Annual observed: 100%	C, D, E, A, U
15 (15)^62^	419 mm (368–470)		10	92	23 m	Maximum: 400 m		None
71 (59)^48^	563 mm (439–868)	3 A (6R)	18	461	30 m	100% at 400 m*	Annual: 31%	C, D, E, A, U
21 (13)^60^	297 mm (297–370)	1 A (20R)	20	205	23 m	Not reported	Obs mean: 135 d	D, E, A
30 (29)^90^	419 mm (310–470)		12	188	40 m	Maximum site 1: 400 m site 2: 200 m	Obs mean: 123 d	C, D, U
60 (34)^41^	404 mm (331–625)	1 A (60R)	60	330	30 m	Not reported	Obs median: 43 d	Predated, E, C, U, tag loss, surface mortality, survival
80 (25)^41^	548 mm (412–761)	1 A (45R)	45	324	55 m	Not reported	Obs median: 42 d	The same as above
85 (71)^x^	505 mm (388–764)	2 A (APS)	49*	534*	38 m	50% at 346 m	Annual: 54%	P, C, D, E, U, A

TL, total length; APS, acoustic positioning system (A, number of arrays; R, number of receivers); DD, deployment days; m, meters. Common fates: C, caught; D, deceased; A, active (survived); U, unknown.

^33^Topping and Szedlmayer, 2011;^34^Williams-Grove and Szedlmayer 2016;^61^Piraino and Szedlmayer, 2014;^59^Williams-Grove and Szedlmayer, 2017;^62^Froehlich et al.., 2019;^48^Everett et al.., 2020;^60^Banks et al.., 2022;^90^Froehlich et al.., (2021);^41^Bohaboy et al.., 2022; ^x^ this study *applies to APS arrays.

Red snapper is a long-lived (> 50 y), highly valued and heavily fished reef fish in the Gulf of Mexico^18,19^ (GOM). The life cycle space use (Table 1) of red snapper is typically considered to be relatively small, with non-overlapping, oceanic adult and juvenile habitats and a resident adult movement type^20^. Red snapper spawn at a wide diversity of sites^21,22^, with most successful larval settlement predicted to occur within 80 km of the spawning site^23^. As red snapper mature (~ age 2 y), they undergo an ontogenetic habitat shift from juvenile habitat to more complex structure, including ARs^24,25^. This habitat preference is consistent for fish ages 2–8 y which are the ages targeted by hook-and-line fishing in high rugosity areas^26^ and commonly studied with AT^17^. A second ontogenetic habitat shift appears to occur in older, larger adults (> 800 mm TL and ~ age 8 y), when they become less attracted to structure and move to deeper, open habitat^17,27^. Dispersal of red snapper at the lifetime and large marine ecosystem scale (i.e., degree of reproductive isolation), remains poorly understood. Genetic patterns suggest sub-populations within the GOM, but their spatial scale remains unresolved^21,28–30^. Similarly, lifetime dispersal is unknown but modeling results^21^ predicted 37.5% of red snapper remain within a 10 km^2^ grid over a generation time (19 y).

Red snapper are currently managed as one stock in the GOM, but with state-specific private recreational fishing sector quotas. The stock was severely overfished starting in the 1960s, underwent a rapid increase in abundance in response to new regulations from 2009 to 2016, and is now growing less rapidly^31^. In the northern GOM approximately 20,000 ARs have been deployed to improve fishing^32^ and red snapper catch rates are estimated to be 20 times higher at ARs than over natural habitat^26^. Red snapper recreational landings in the GOM are highest off Alabama and the Florida panhandle, where there are extensive ARs^26^. Given the high fishing effort at ARs and AT results suggesting high site fidelity to ARs^33,34^, management was interested in testing if there was connectivity between natural and artificial reefs to determine if they could designate some ARs as fishing zones, with only local effects.

**Fig. 1:**
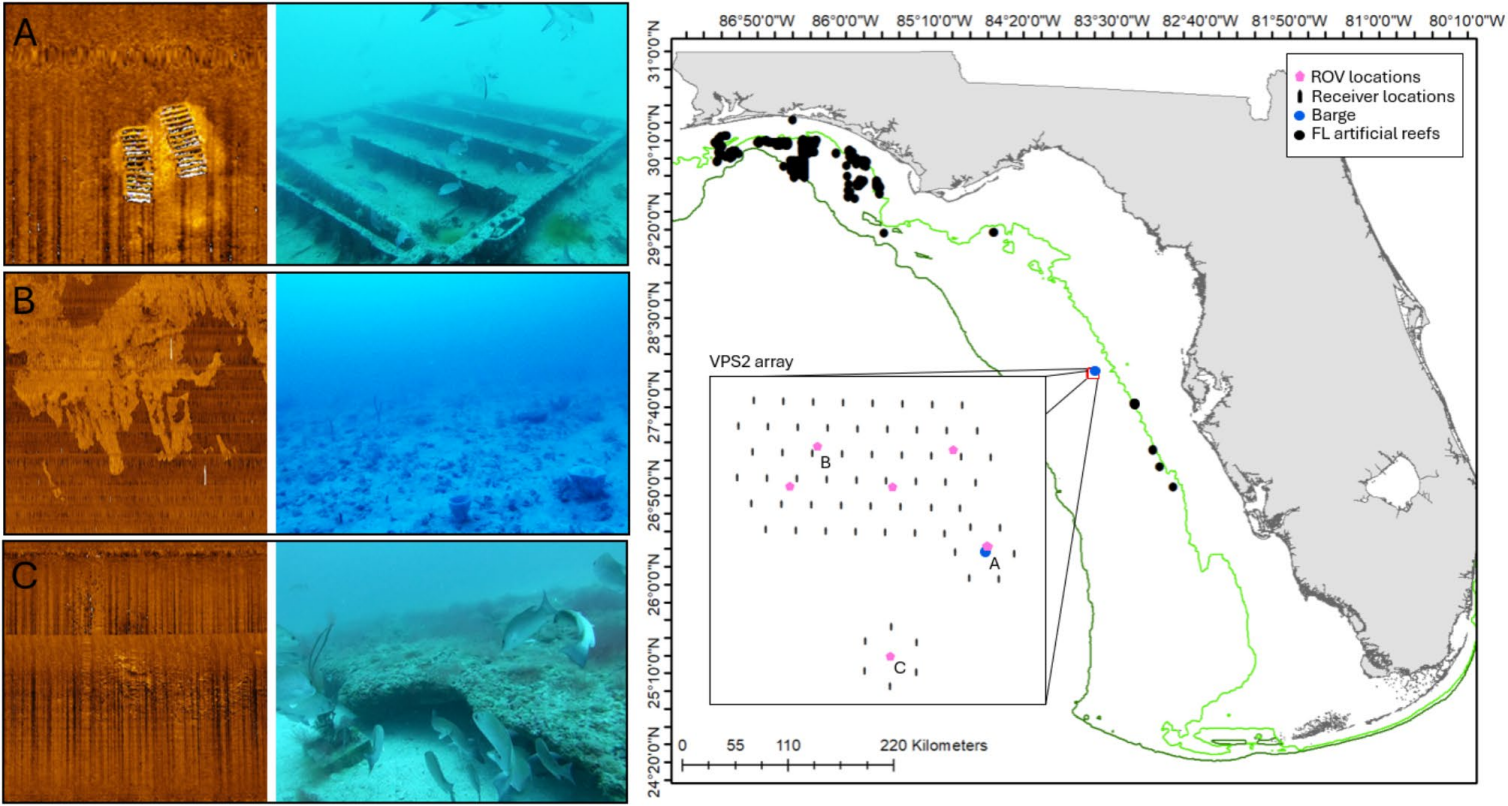
Map of the west Florida shelf indicating the location of the study site (red box), the 30 m and 90 m depth contours (green), and permitted public artificial reefs in 30–90 m water depths (where red snapper occur), off the west coast of Florida. The inserted box shows the second APS (APS2) array design and sites surveyed with video for fish abundance (pink markers). The three habitats monitored include: (**A**) the artificial reef (sunken barge); (**B**) example hard bottom site; and (**C**) the ledge. Images from the ROV surveys and side scan sonar for each of these habitats are shown to the left.

**Fig. 2:**
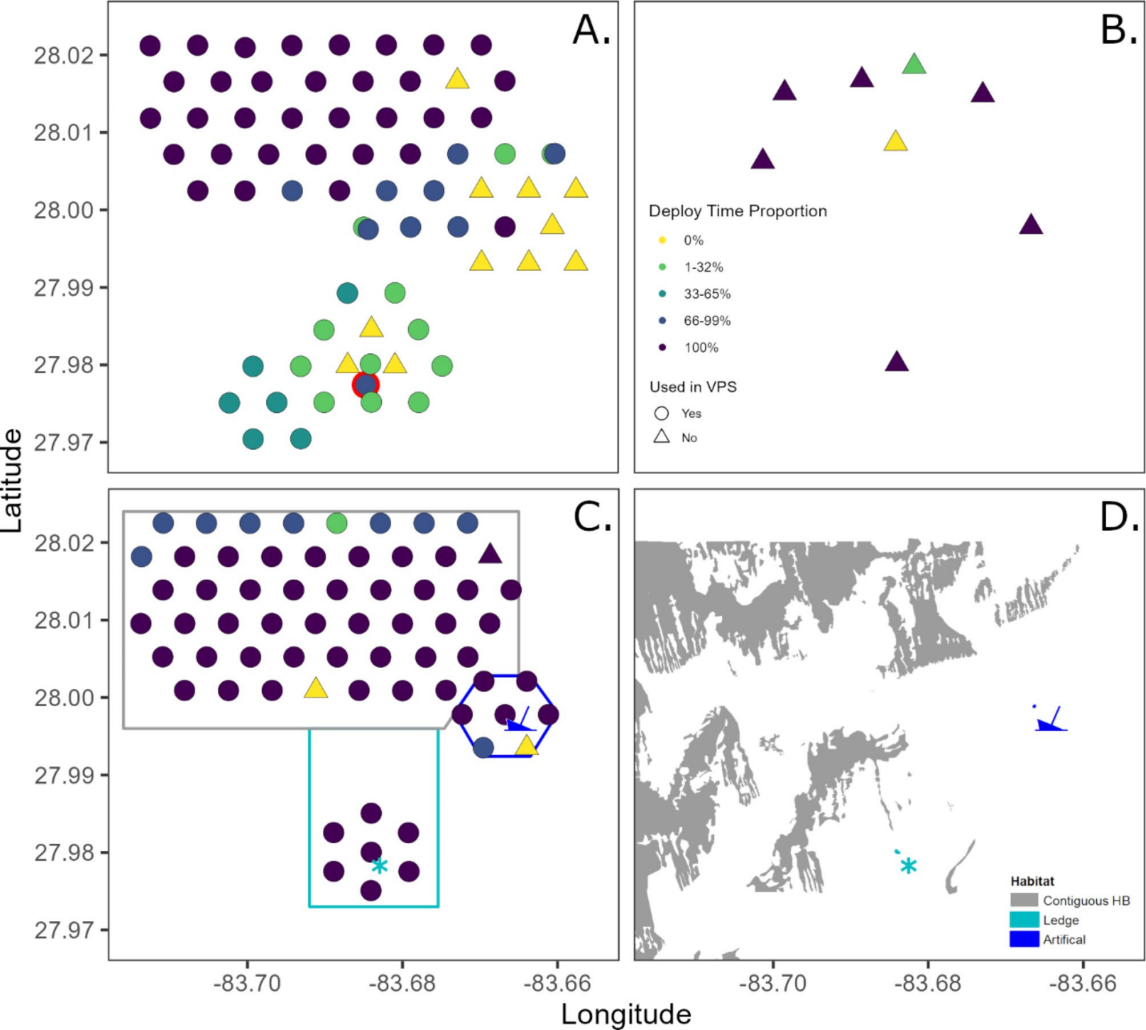
Three receiver arrays were deployed during this study: the first large-scale acoustic positioning system, opportunistic data was retained from one moved receiver, indicated by the red circle (APS1;** A**), an interim array to monitor tagging sites (**B**), and a second large-scale APS (APS2; **C**), with habitat-specific array components indicated by: grey box = hard bottom, royal blue hexagon and ship = the artificial reef, teal box and asterisk = ledge; and the habitat mapped by sidescan; (**D**). Receiver markers are color-coded to indicate the proportion of realized versus expected days of data collected due to disturbance from shrimpers. 0% indicates a receiver was not retrieved or malfunctioned.

To answer these questions, we designed a study off western Florida, in an area with relatively few public ARs (Fig. 1). The study area encompassed ~ 12 km^2^ and included a natural reef ledge, extensive low-relief hard bottom (HB), and an AR. Data were collected with multiple methods (traditional AT, a mobile tracking platform (glider), video data collected by ROV, and a dart tag recapture hotline). The original array design was a large, 69-receiver APS (APS1). However, unexpected shrimp trawling in the area impacted receiver location and retrieval, resulting in the need for an interim presence/absence array at tagging sites, followed by the deployment of a second 60-receiver APS array (APS2; Fig. 2). These challenges resulted in a complex data set and the need to think through how changes in receiver coverage affected inference power, as well as the location accuracy needed for each movement metric (Table 1).

We test multiple hypotheses about red snapper movement metrics, their relationship to habitat, and our ability to accurately estimate them. Fate assignment greatly affects inference power^35^ (Table 1), given the need to censor detections from dead fish, analyze detections only of the study species, and determine emigration events. We test the hypothesis that fate, especially emigration from the study area, could be accurately assigned, and we evaluate the effect of unknown fates and model selection on estimates of site fidelity. To better understand fidelity to our study area, we estimate multiple residence times: (1) at 50% site fidelity to be comparable to previous studies; (2) 40% site fidelity to compare to that expected over a generation time^21^; and (3) 1% site fidelity to estimate population replacement time. Site fidelity and space use often vary with tracking duration^36^, sensitivity to proximate cues such as hurricanes moving through^37,38^, attraction/avoidance of certain habitats, and individual “personality” (i.e., genetic predisposition to exploratory behavior, stayers versus movers)^39–41^. To explore these drivers of space use and emigration, we test if space use significantly increases with tracking duration and habitat patch size, and if emigration rates increase with storm fronts. Lastly, we test the hypothesis that differences in inference power between this study and in previous red snapper AT studies is minor, allowing us to compare results of site fidelity, space use, and dispersal across studies. results from previous studies.

## Results

The study area was 101 km offshore and included three habitat types (HB, ledge, and AR) with variable patch size. A total of 3,518,071.8 m^2^ of HB was mapped with side-scan sonar. The APS arrays monitored 460,336.9 m^2^ of this, whereas the ledge and the AR had small footprints (1,719.2 m^2^ and 425.7 m^2^, respectively) that were fully monitored by the APS. Variations in natural noise across habitat types affected detection range and location accuracy. Mean (± standard deviation throughout) daily environmental noise at 69 kHz was greater over natural structure (456 ± 138.6 mV over HB, 314 ± 73.3 mV at the ledge compared to the AR (269 ± 55.7 mV).

**Fig. 3:**
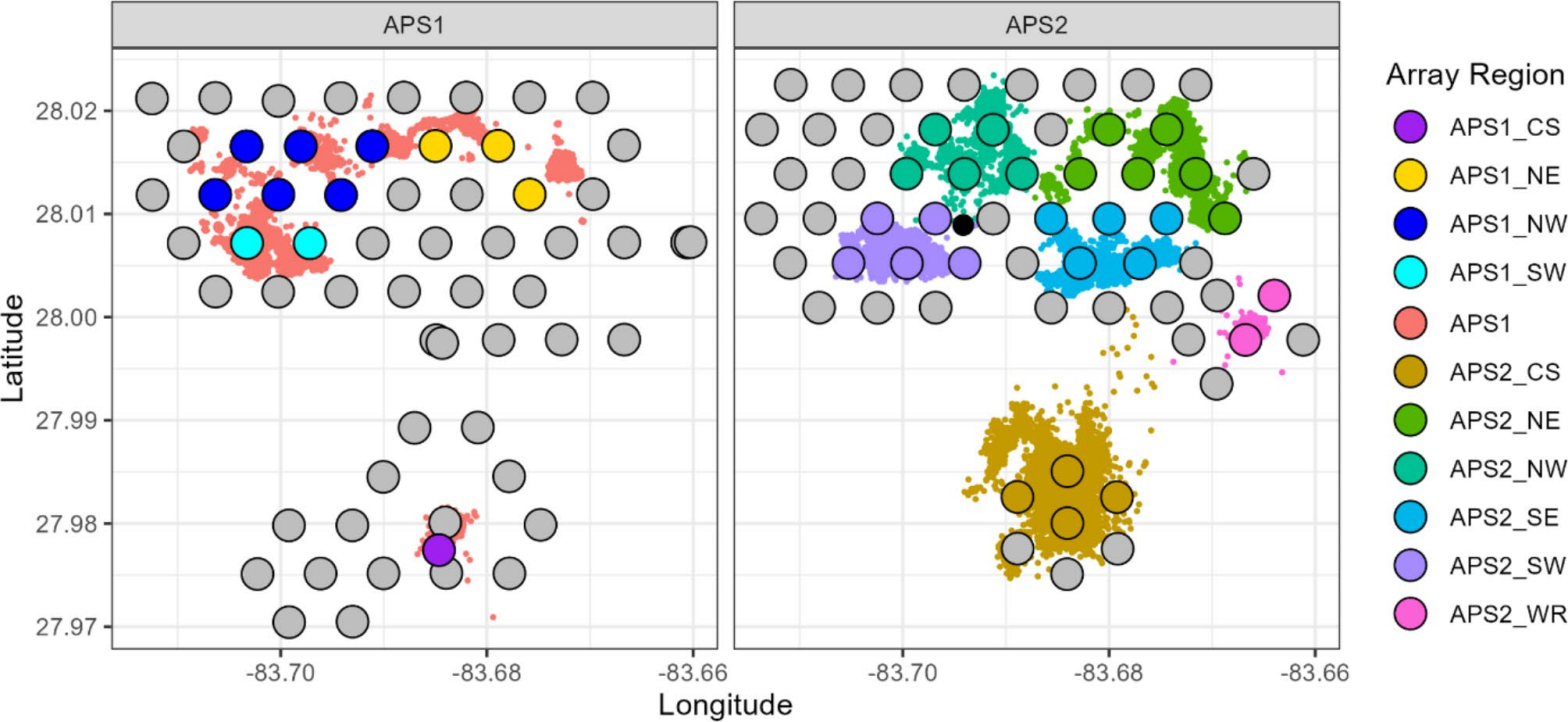
Receiver locations in both acoustic positioning system arrays (APS1 and APS2) and all Innovasea-provided fish positions. Known position error from color-coded receivers was used to evaluate if one or more position filtering relationships was needed, i.e., did position error vary within the array. The reference tag location in APS2 is denoted by a black circle.

A lower-than-expected detection range in both APS resulted in variable and often relatively large time steps between estimated fish positions. As a result, we use both positions and COAs, depending on whether high location accuracy or continuous detection coverage, respectively, was needed. We had expected to estimate 50% detection range using the array’s sync tags. These are tags internal to the Tx receivers used to synchronize receivers’ time clocks for accurate positioning. The 50% detection range of our sync tags was 704 m in APS1, and 697 m in APS2, very close to the expected range of ~ 700 m. However, the 50% detection range of our reference tag placed over HB (Fig. 3) was 346 m, with a maximum detection range of ~ 1700 m. The key difference between the reference tag, which had the same specifications as tags implanted in fish, and the sync tags was the addition of a pressure sensor.

This was much lower than the position error threshold (20 m) resulting in 86% position retention when we applied post-processing position filtering. The filtering process uses one or more relationships between known position error, i.e., hyperbolic positioning error (HPEm, m) and Innovasea-computed estimates of position error sensitivity (HPE, unitless) to filter fish positions that were based on less than ideal triangulation conditions. Here we used sync tags in locations overlapping with fish positions to evaluate if position accuracy varied within the array, which would necessitate more than one filtering relationship (Fig. 3). A single relationship was sufficient for the APS1 array (Figure S1; Table S1), but not for APS2 (Table S1). To standardize position accuracy throughout both APS arrays, the position accuracy threshold was set to 20 m. The median horizontal location error for the reference tag was 3.64 m (n=55,775 detections). For 30-minute COAs it was 164 m (*n* = 7,554), and 149 m for four-hour COAs (*n* = 945); Figure S2). Although this location error is much higher than the 20 m assigned to positions, it was adequate for answering research questions about fate and site fidelity. A total of 11,398,890 red snapper detections were collected, resulting in 745,125 30-minute COAs. A total of 889,218 positions (11% position yield) were provided by Innovasea. After filtering for position error, 711,723 positions were retained for 66 fish. Ten fish had low position yields and did not satisfy the minimum data requirements to be included in our estimates of space use.

**Fig. 4:**
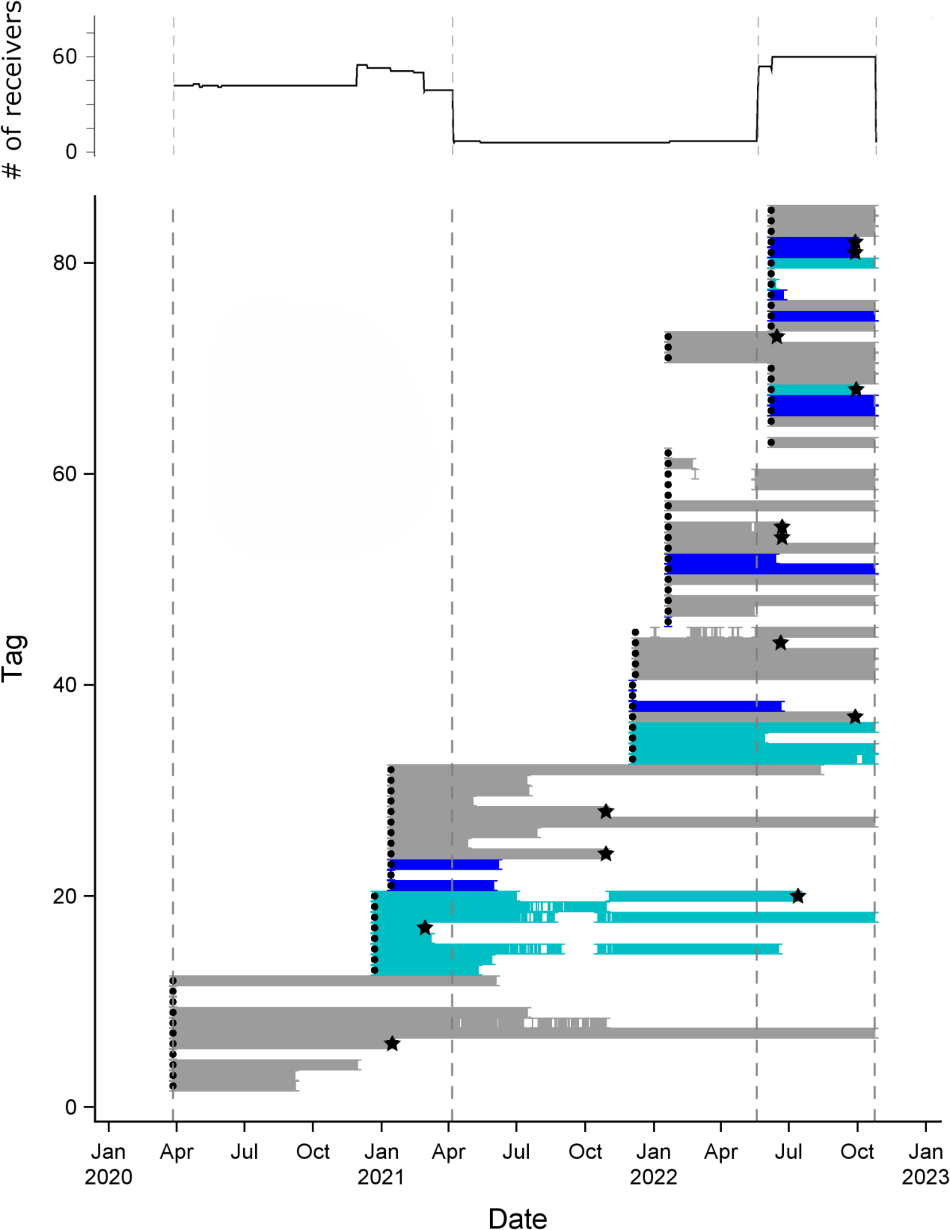
The number of functional receivers by date (top) and detection dates of fish by tagging location (bottom). The first two dashed lines represent APS1 deployment dates and the second two dashed lines represent APS2 deployment dates. Tagging habitat is grey for hardbottom, teal for the ledge, and blue for the artificial reef. Tagging date is denoted by a dot and fish that emigrated are denoted by a star.

In total, 85 red snapper were acoustically tagged over four tagging periods (Table S2, Fig. 4) with a mean size of 505 mm TL ± 69.7 mm TL. Seventy-one of these fish were tracked after the tagging recovery period (3 d): 12 tagged at the AR, 44 tagged over HB, and 15 tagged at the ledge. The mean number of functioning receivers for both APS was 49 (Fig. 4). The area monitored, number of days an array was deployed, and the number of fish tracked during that time varied with each array. APS1 was 15.7 km^2^, deployed for 375 d and monitored 27 tagged fish. The mean potential tracking duration of these fish (based on tag date and APS1 recovery date) was 177 d. APS2 was 12.8 km^2^, deployed for 159 d and monitored 48 tagged fish that had a mean potential tracking duration of 125 d. The interim array was deployed for 405 d between APS deployments and monitored 45 fish. Two tagged fish were never detected, and another two fish had malfunctioning tags (recaptured by staff in the array 186 d after their last detection).

*Fate assignment.* Predation was relatively high during the tag recovery period and it was not always possible to identify emigration. Ten fish died during the three-day recovery period: three of unknown cause and seven predated. For the remaining 71 fish we assigned the following fates: 8% mortality (of unknown cause), 9% capture removal, 45% survival (remained alive and detected within the study area), 3% predation (assumed based on movement signatures unlike those of red snapper), 21% emigration, and 14% unknown (detections stopped and detection and recapture data were insufficient to assign a cause). Predation was based primarily on large horizontal movements not seen in red snapper. Most of the unknown fates (70%) occurred during the interim array period when it was difficult to observe these movements. Percent agreement in fate assignment among three scientists was high (93%), with recaptures (*n* = 5 fish) and glider detections (*n* = 2) playing a critical role in identifying and confirming emigration events. Tags leaving the array (both unknown or emigrated fates, *n* = 25) were highest in May or June (*n* = 12) when temperatures warm, spawning starts, and the recreational fishing season opens; and also in September or October (*n* = 8) when storm fronts were more common (Fig. 4).

### Site fidelity

The mean residence index of tracked fish during the study was high (97% ± 9%, *n* = 71). APS array overlap with fish space use resulted in very few daily detection gaps (1 d gap, *n* = 2 fish; 8 d gap, *n* = 1 fish). Not surprisingly, daily detection gaps were more common in the interim array (1 to 125 d gaps, *n* = 10 fish). The mean tracking potential duration for all fish (*n* = 71), based on when fish were tagged and when arrays were removed was 427 d ± 269 d. Mean observed residence was roughly half of this, 246 d ± 170 d (range: from 7 d to 942 d, *n* = 57). For fish exhibiting fidelity to their tagging habitat, mean realized versus potential residence times varied by habitat, with the highest observed residence times occurring over HB (Table 3).

**Table 3:** Mean realized and potential residence times (days) by habitat type and standard deviation (sd).

Habitat	*N*	Mean realized residence time (sd)	Potential tracking duration (sd)
Ledge	8	141 (92.5)	386 (249)
Hardbottom	29	258 (179.0)	385 (255)
Artificial reef	3	140 (0.0)	140 (0.0)

Emigration increased on days with low atmospheric pressure and annual fidelity estimates to the study area were affected by model choice (empirical or parametric) and treatment of unknown fates (Table 4). The daily emigration probability model was run with fish that had unknown fates censored on their last detection day. The most parsimonious model was one that included only atmospheric pressure as a predictor of daily emigration probability (Table S4), and it estimated that this probability was about 175 times higher on days with lower pressure than on a regular pressure day (Table 5). Annual site fidelity estimates based on the commonly used empirical Kaplan-Meier estimator were lower than those estimated with the parametric Weibull model (Table 4), and unknown fate treatment (considered as emigrated or censored) decreased results by approximately 20% for both models. Assuming fish with unknown fates represent a mixture of fish that emigrated as well as were captured and predated we consider the mean of the treatments the most representative estimate of annual site fidelity: 54% (empirical method) and 63% (parametric model). Mean residence times of interest were as follows: 496 d (50% of fish); 609 d (40% of fish) and 5.6 y (1% of fish; representing population replacement).

**Table 4:** Annual site fidelity and residency values for the nonparametric Kaplan-Meier estimator and the parametric (Weibull) model for treating unknown fish as emigrated or censoring them.

Model	Fates	Number emigrated	Estimate (CI)	50% residence	40% residence	1% residence
Empirical	U = E	21	0.48 (0.29–0.67)	300 d	380 d	-
	U = P, C	11	0.60 (0.38–0.81)	-	-	-
Parametric	U = E	21	0.55 (0.27–0.66)	408 d	518 d	5.6 y
	U = P, C	11	0.71 (0.33–0.82)	583 d	700 d	5.5 y

E , migrated; P, predated; C, capture removal.

**Table 5:** Predicted marginal effects of atmospheric pressure on daily emigration probability on the inverse logit scale.

AtmP	Estimate	Confidence interval	
Low pressure	0.07198	0.03415	0.12968
Regular pressure	0.00041	0.00017	0.00081

*Space use and movement patterns.* Red snapper abundance and space use varied with habitat type, but did not increase as expected with tracking duration or habitat patch size. Video-based abundance estimates per sampling event were fairly low and highly variable, with a mean count of 4.5 ± 8.0 red snapper. However, abundance was higher at the habitats with small spatial footprints, regardless of whether they were natural or artificial (AR: 7.1 ±- 6.8 fish; ledge: 14.2 ±- 13.6 fish; and HB: 1.6 ± − 1.1 fish). Median space use over the study duration, based on the 95% utilization distribution, was 0.039 km^2^. This was much smaller than the area monitored by the APS arrays (≥ 12 km^2^) and it did not significantly increase with tracking duration as expected (ρ=-0.26174, *n* = 56, *p* = 0.0513). However, space use over the duration of the study did differ by habitat. It was intermediate over HB (0.029 km^2^), smallest at the AR (0.017 km^2^), and largest at the ledge (0.067 km^2^) and these differences were marginally significant (quartile regression, *n* = 56, χ^2^ = 0.0441).

**Fig. 5:**
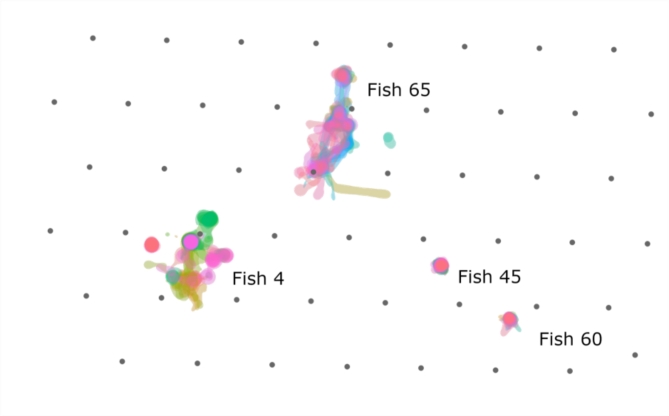
Although median daily space use was smaller than tracking duration space use for all fish, the amount of overlap in daily space use varied. Here we show the individual variability in daily space use based on 95% utilization distributions (colored by day) for four fish over hard bottom. The degree of daily space use overlap was not correlated with tracking duration: fish 4: 248 d, fish 65: 140 d, fish 45: 322 d, and fish 60: 278 d.

**Fig. 6:**
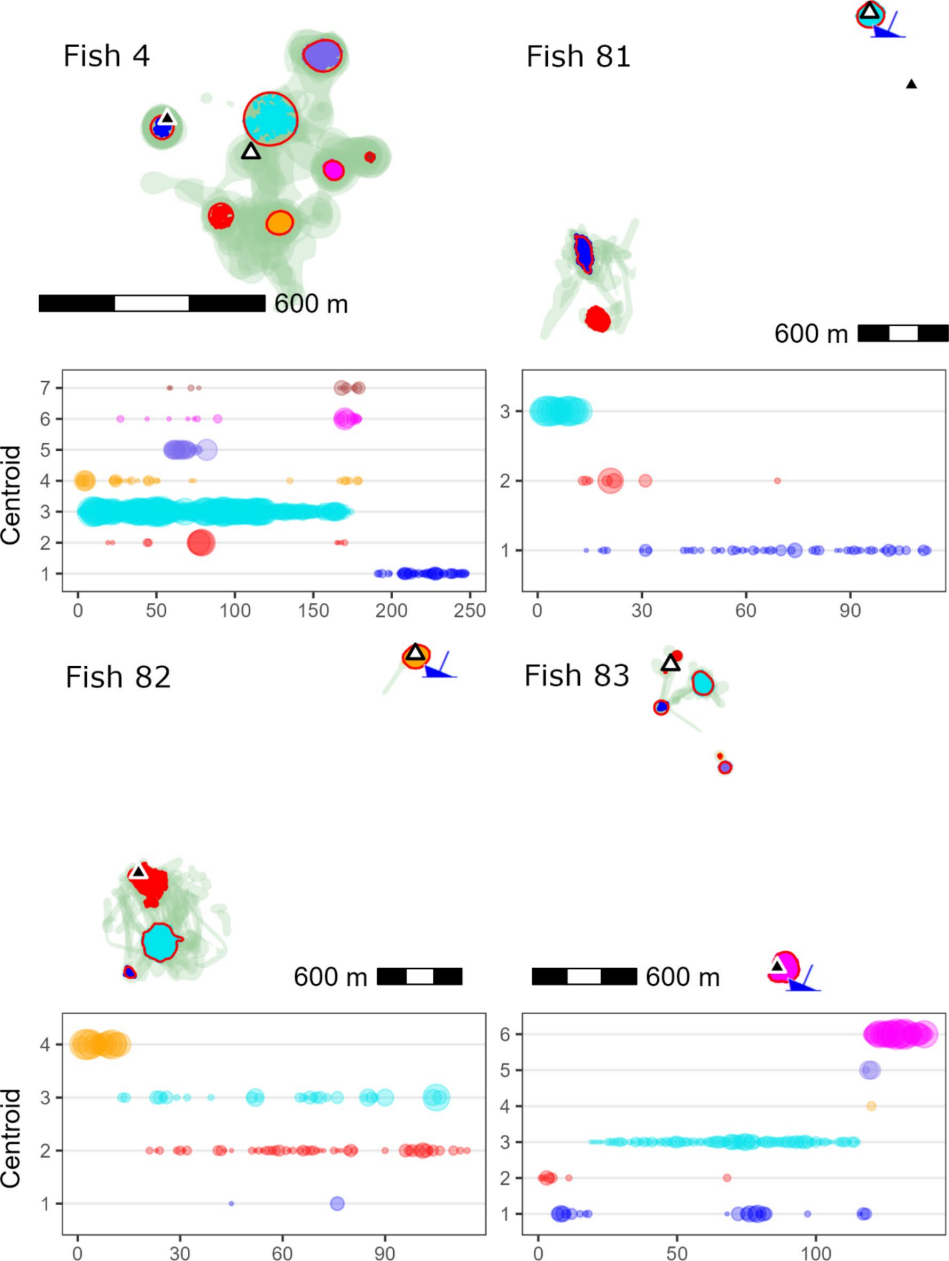
Individual space use differed in terms of how many high-use sites (centroids) occurred and the temporal use pattern of centroids (90% UD contours and a minimum size of 100 m^2^) within tracking duration space use. Four fish exhibited complete shifts in space use. Fish 4 remained over hard bottom but shifted its use to the west. Fish 81 and 82 shifted from the artificial reef (boat icon) to the ledge, and fish 83 shifted from hard bottom to the artificial reef. Tagging locations are indicated by white triangles and final positions by black triangles.

Daily space use was considerably smaller, but also varied with habitat type. Daily median space use was largest at the ledge (0.026 km^2^), but similar over HB (0.012 km^2^) and the AR (0.014 km^2^). The degree of daily space use overlap varied across individuals (Fig. 5). Some fish had their daily detections concentrated at the same location (centroid) over the study period while others shifted centroids over time, with a mean of 2 +/- 1.4 centroids per fish (*n* = 56, range: 1 to 7). Most fish that shifted centroids continued to have intermittent periods of detection at previous centroids, but four moved to new, non-overlapping locations (Fig. 6). Three of these moved either to or from the AR.

**Fig. 7:**
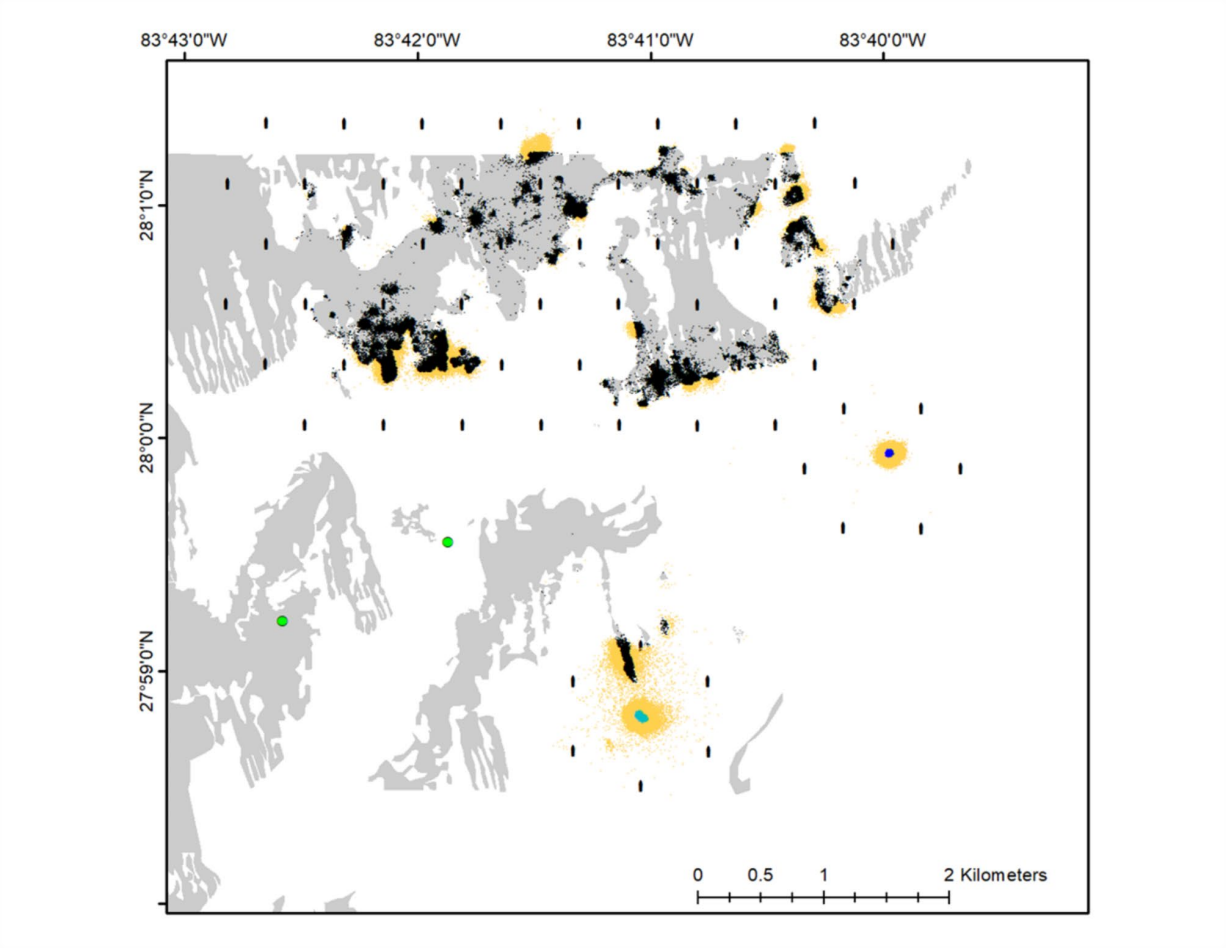
Fish positions (with ≤ 20 m position error) from both acoustic positioning arrays categorized by underlying habitat: black = hard bottom, royal blue = artificial reef, teal = ledge, and yellow = sand. Green dots represent live red snapper that left the array but were detected by the glider over nearby hard bottom.

**Fig. 8:**
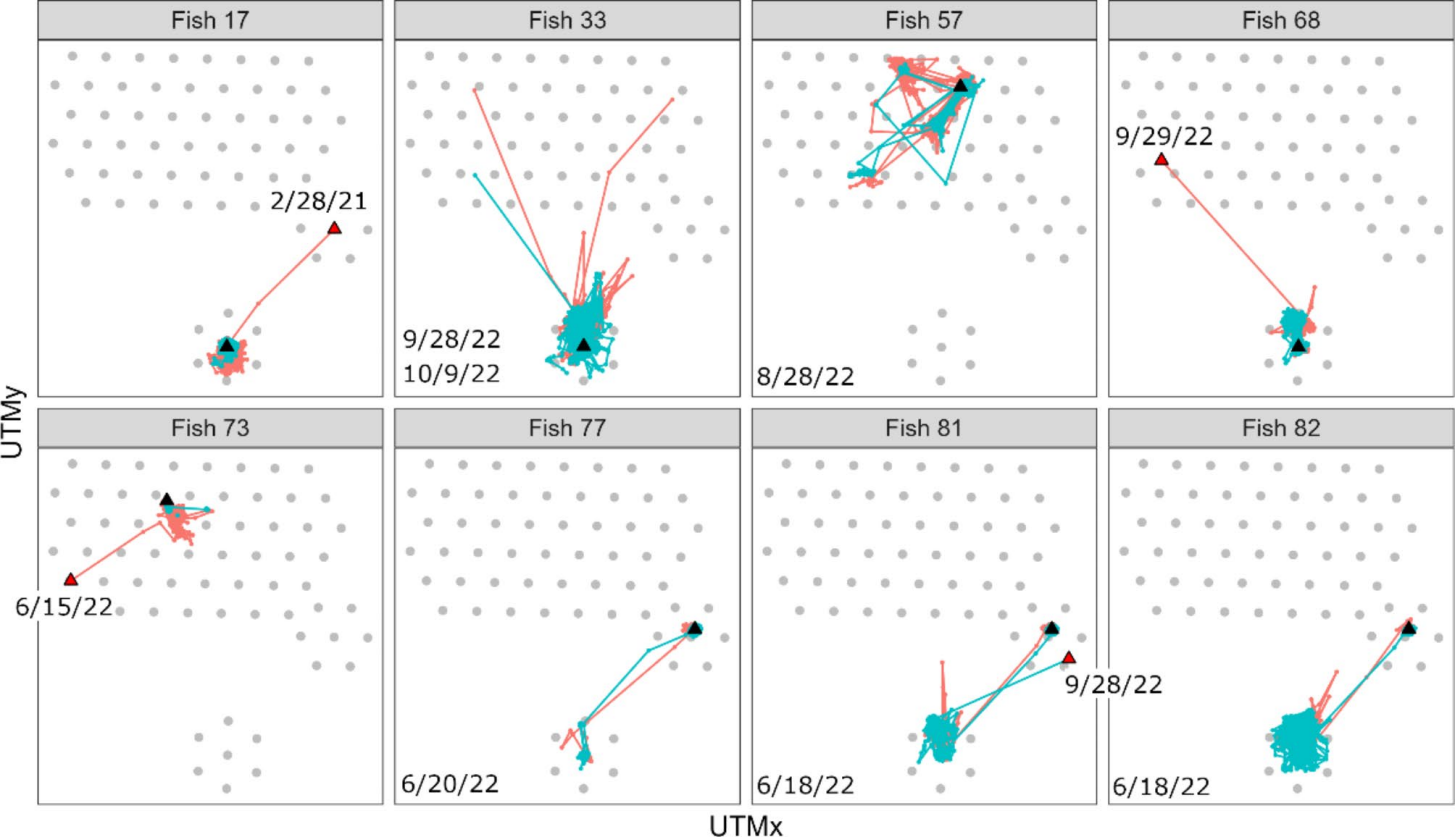
Long distance movements (LDMs) and APS2 receiver locations. Movements based on positions are in teal and those based on 4 h COAs are in red. Fish 33, 77, and 81 made two LDMs. Tagging location is indicated by black triangles and LDMs associated with permanent emigration from the array are indicated by red triangles shown next to emigration dates. LDMs not associated with emigration have LDM dates noted in the bottom left corner of each panel.

Most observed movements were small, with relatively slow movement rates, and over habitats with structure. However, 18% of positions occurred over sand (Fig. 7), and rapid, long-distance movements that included moving over sand were also observed. The median distance between positions was 10.3 m and the median movement rate was 0.03 m/sec. However, 13 fish made movements with a rate more rapid than the maximum previously reported for this species (> 0.75 m/sec) and two of these fish were confirmed as red snapper based on later recaptures. Detection probability of ephemeral long-distance movements is lower than that of small, slow movements, and more long-distance movements were identified with COAs than positions. Of the 71 tracked fish, 17 made long distance movements, many of which were associated with leaving the array. However, 8 fish made within-array long distance movements (Fig. 8) and these movements occurred over HB and between habitat types. One fish left the ledge, moved over HB, was outside of range for 8 days and then moved over the HB habitat to return to the ledge. Within-array long-distance movements were most common in June 2022, overlapping with when the red snapper fishing season began. The for-hire recreational fishing season opened on June 1, 2022, and the private recreational fishery on June 17, 2022. Two confirmed capture removals occurred during this time, one at the ledge and one at the AR.

### Dispersal

**Fig. 9:**
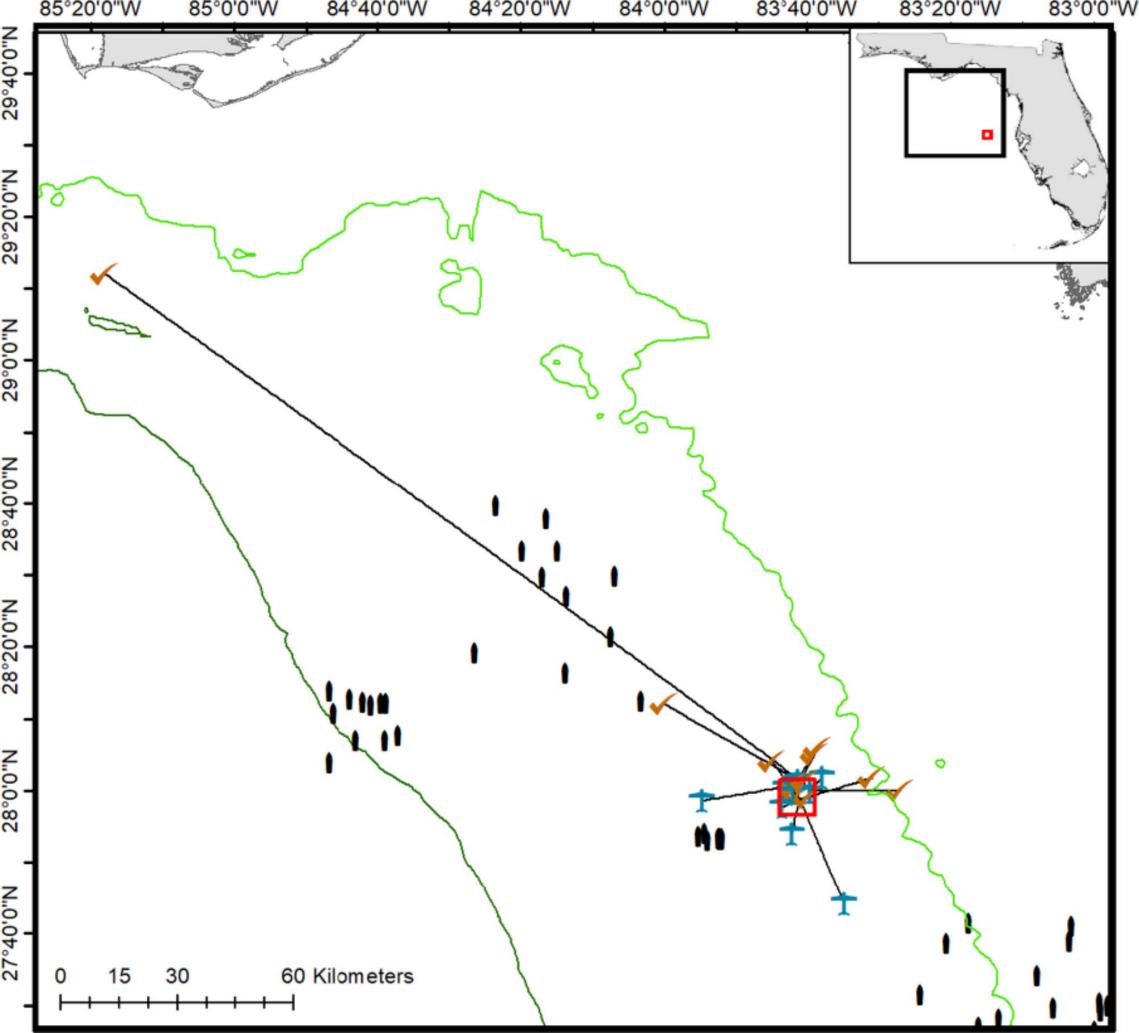
The study area (small red square) versus dispersal area. Dispersal from the study area was detected either by the glider (plane icon) or by hook-and-line recaptures (check mark). Receivers belonging to arrays within the iTAG regional telemetry network in the dispersal area are indicated. Straight lines indicated how emigration path distances were estimated. Green contours indicate water depths of 30 m and 90 m.

Although most fish dispersed < 2 km (*n* = 66), individual dispersal was highly variable ranging from 0.5 km to 206 km and occurred in all directions (Fig. 9). Five fish dispersed > 20 km, but were not detected in other iTAG Network arrays, even though several were deployed in adjacent areas, including one 23 km SW of the study site. The Burr model had the most parsimonious fit to the dispersal data (lowest AIC, highest loglikelihood). For a mean observation period of 297 d ± 213, it predicted a 31% probability of a tagged red snapper moving ≥ 2 km, a 10% probability of moving ≥ 10 km, and a 1.2% probability of moving the maximum distance observed (approximately 200 km; Figure S3).

### Inference power of previous AT studies

In nine previous red snapper AT studies (Table 2), the mean number of functional receivers was 27 +/-16.9 (range: 10 to 60). Seven studies used APS arrays, most of which were small (0.25 km^2^ to 2.77 km^2^), with the most common approach being the use of one to six small APS arrays made up of 5–6 receivers (approximately 1.39 km^2^). However, one study deployed two large APS arrays (approximately 15 km^2^ each) for 324 d and 330 d, respectively. Study duration also varied, with deployment days ranging from 188 to 1,639 d. The number of fish tracked in each study ranged from 7 to 59, with the mean size of fish ranging from 297 mm to 639 mm TL. All studies tracked fewer fish than they tagged. For studies with a tracking duration > 200 d, the proportion tracked to tagged ranged from 56 to 83%, with the lowest proportion due to high tag loss of externally attached tags.

## Discussion

Past AT studies have focused primarily on young adults (≤ age 8) at ARs in the northern GOM, creating a mismatch between the spatio-temporal scale of data collection, the red snapper lifespan (> 50 y), and range of habitats used^33,34,41,48,59–62,90^. This study increased our understanding of habitat use, but like previous studies focused on young adults. Approximately half of the young adult red snapper tracked in this study used the same general location within a given year for foraging, spawning and overwintering, which is uncommon in marine fishes^1^, but may be possible due to the diverse diet of red snapper, including zooplankton^42,43^. We observed larger space use than past studies, complex fine scale movements over HB, connectivity between artificial and natural habitats, and highly variable dispersal. We also estimated a replacement time of 5.6 y, which is relatively short given the species’ lifespan.

Because of how AT works, results are affected not just by behavior but by inference power. Our APS arrays were designed for a consistent range over space and time of approximately 700 m. The realized range was approximately half of that and varied within APS region. This resulted in relatively low position yield, higher than expected position error, and relatively large and variable time steps between positions. As a result, we needed to refine the research questions we could answer with these data and use both positions and COAs. Shrimping in the area also caused modifications to the planned arrays, and more importantly, the need for an interim presence/absence array. Because of the low receiver density of the interim array it was more difficult to assign fate during this time period, there were larger daily detection gaps, and we could not estimate space use. The complexity of the data due to these challenges led to us develop the concept of inference power. We hope that by being transparent about the challenges we faced and how we addressed them, other scientists might not need to repeat our mistakes. In addition, given that many of these challenges are common to AT studies^11^, if we want management to more fully use AT results, we need to develop the means to measure uncertainty in these results and develop standards for reporting it. Below we discuss fate assignment and our movement trait results in the context of prior red snapper AT studies, factors affecting inference power, and make suggestions to improve reporting and inference power in future studies.

Incorrectly assigned fates in AT studies can bias results^44,45^. In this study we used pressure sensors to track water column use, a large array that could track extensive horizontal movements, and auxiliary data (recaptures and glider detections), but still needed to assign an “unknown” fate to some fish. Most fish in this study left the study area during the interim array, but unknown fates also occurred during APS arrays when fish located near the edge of the array stopped being detected. We initially planned to use a threshold movement rate to assign predation^46^, but we observed some confirmed red snapper movements as fast as those reported for the predators. Fates were assigned in all but one of the previous red snapper studies, but fate categories and criteria to assign them were often unclear and varied. Several authors have reported an unknown fate category^33,34,41^, but did not report how this affected results. Here, the inability to distinguish between emigrated, predated, or captured red snapper resulted in approximately a 20% difference in estimates of annual site fidelity to the study area.

Accurate residence time and site fidelity estimates are critical to understanding vulnerabilities to spatially explicit stressors and efficacy of marine protected areas, and there is a need to more fully standardize definitions and methods to estimate these important metrics. Median reported residence times at ARs from previous red snapper AT studies range from 43 d^41^ to 23 mo^34^. However, Bohaboy et al.^41^ report observed median residency and estimated residence time at the release reef^41^ whereas Williams-Grove and Szedlmayer^34^ considered residency to include short-term movements to nearby ARs and defined median residence time as the time period when 50% of the active tagged red snapper were still present after three years.

Here we evaluated residence and site fidelity at the scale of our APS arrays (12.8 km^2^ and 15.7 km^2^). We used the residence index (mean = 97% ± 9%) to show that our APS arrays overlapped well with fish space use, with few daily detection gaps. Mean observed residence times (246 d ± 170 d) were roughly half of the mean tracking potential duration (427 d ± 269 d), indicating many fish left the study area before the study ended. Although residence times are often thought to be longer at ARs than over natural habitat, the longest observed residence time in this study (258 d) was over HB. Most tags that left the array did so in May or June; or the left in the fall. The early summer emigrations may have been associated with recreational fishing activity^47^ and the fall emigrations were associated with storms and decreased atmospheric pressure which increased the probability of emigration.

Event history analysis, where emigration is the event, was used to estimate annual site fidelity to the study area and residence time (i.e., time associated with a given proportion of fish remaining in the study area). AT studies typically use the non-parametric Kaplan-Meier estimator^33,34,48^ to calculate annual site fidelity and 50% residency. This method is relatively simple and does not assume any specific underlying distribution for site fidelity times. However, since it does not estimate any parameters, it cannot be used to extrapolate beyond the range of the observed data. To do so, we used a parametric (regression) model. This type of model can be sensitive to the assumed underlying distribution, necessitating an evaluation of different distributions and model comparison to select the best. Annual site fidelity ranged from 48 to 71%, with estimates affected by the method used (parametric vs. non-parametric) and how fish with unknown fates were treated (Table 4). Results which most closely resembled mean observed residence time were from the empirical method, when fish with unknown fates were assumed to have emigrated (300 d vs. 246 d).

Our mean empirical method results of 54% annual site fidelity fall within the range of results from previous red snapper studies (31–82%; Table 2) that used the same method but monitored smaller sites (one to two ARs), with smaller APS arrays (5–6 receivers). The lowest annual site fidelity estimate (31%) is from a recent study tracking red snapper at oil and gas platforms^48^, and the two highest estimates (72% and 82%, respectively)^33,34^ are from earlier studies, when red snapper abundance was lower and there were more restrictive fishing regulations. These differences could reflect differences in red snapper behavior, especially if emigration increases with fishing effort. But due to variable inference power, it was not possible to rule out that these differences are due to observation error. Studies differed in array size, array deployment days, fates assigned, and tag recovery period (Table 2). The impact of tag recovery period is highlighted in Topping and Szedlmayer^33^ who report annual site fidelity was reduced from 72 to 61% if fish that emigrated early (< 6 d) were not censored. In addition, the study with the lowest site fidelity^48^ also had fairly high daily detection gaps, suggesting that the array might not have been large enough to monitor shifts in daily space use, and these were assigned as emigrated.

We recommend future studies report site fidelity and residency definitions, criteria for assigning a fate of emigration, array area monitored, daily detection gaps, and potential tracking durations. Given a lack of data on how long a fish was in residence prior to capture and tagging, annual site fidelity and residence times are always under-estimated. However, factors affecting potential residence time such as tagging dates and array deployment dates are known and need to be reported to accurately interpret observed residence times^49^. It is also important to recognize that residence times will not be invariant over time. Both density-dependent effects^50^ and changing environmental cues^36^ will affect residence times and both are expected to change over time, as the red snapper stock recovers and weather patterns change.

Previous research on young adult red snapper suggests small space use^41,51,52^ and greater abundance at ARs than over natural structure^26,53,54^, due in part to the small footprints of ARs^55,56^. In this study we observed greater abundance at sites with smaller footprints, but it included both the natural ledge and the AR, similar to recent results in Bacheler et al.^57^. In addition, space use is affected by habitat context or the landscape within which a given site is placed. Here we observed large space use at the ledge, apparently due to the proximity of additional HB to the north of the ledge. In addition, roughly 50% of the fish tracked over HB exhibited space use similar to or less than the maximum space use seen at the artificial reef, suggesting not all HB is equal, and red snapper have an affinity for “sites” unrecognizable to us.

Daily space use was much smaller than space use over the duration of the study, with some fish consistently using the same space over time while others used multiple centroids. Although the smallest daily space use observed in this study occurred at the AR, our estimate was approximately seven times larger than that reported in Bohaboy et al.^41^ (median space use = 1,890 m^2^). The cause of this difference is difficult to interpret as both studies had similarly large APS arrays and numbers of fish tagged, and the differences in space use cannot be fully explained by differences in position error threshold (Tabe S3). A potential explanation is habitat context, i.e., there was no nearby HB in the Bohaboy et al.^41^ study.

There are tradeoffs between high resolution fine scale tracking versus being able to track fish over larger spatial scales. Array size and data filtering can bias results towards smaller movements and lower movement rates. For example, Bacheler et al.^51^ excluded individual movement rate estimates for detections > 20 min apart and consequently periodic large movements of fish. This may have resulted in movement rates for some fish to be biased low. It has been suggested that reef fish may avoid moving over sand^50,58^. In this study we observed long-distance movements over sand, connectivity between habitats, and higher maximum movement rates than previously reported for red snapper^46,51^. These larger, more rapid movements were less common than small, slow movements, may be cued by disturbance events, and are also less likely to be detected.

We often could not compare our space use estimates at the tracking duration scale to those of previous red snapper AT studies, as reported estimates were volume-based^59,60^ or relative change with season or time of day rather than absolute numbers^34,61^. However, of the two studies that report horizontal space use over the study duration, our median of 0.039 km^2^ was larger than Everett et al.^48^ (maximum 95% KDE of 0.020 km^2^) and smaller than Froehlich et al.^62^ (mean 95% KDE of 0.078 km^2^). But again inference power differed, making it difficult to exclude the potential for observation error. Everett et al.^48^ used small APS arrays made up of six receivers (Table 2), which would limit space use estimates, and Froelich et al.^62^ used 20-minute COAs with a 2-receiver threshold, which would have had a high location error. We recommend several simple steps to improve our ability to compare space use across studies: reporting 95% horizontal space use even in studies evaluating volumetric space use, estimating space use at both the daily and tracking duration scales, assessing the accuracy of the chosen location estimator, and reporting the area of array coverage, as well as daily detection gaps.

Dispersal drives population connectivity and structure, often has a genetic basis^63^, and links to other traits such as sensitivity to environmental conditions^64,65^. Although AT networks improve the spatial scale over which AT studies can track tagged fish, the area over which fish can move (especially those using deeper water) versus the area monitored with receivers makes it difficult to estimate dispersal distances using only AT. As a result, AT studies need to integrate other data sources such as capture data, but with the recognition that angler-reported locations may have lower accuracy and fishing effort is rarely evenly distributed^26,66^. The range of observed dispersal in this study was similar to those reported in traditional tag/recapture studies in the northern GOM^67–69^. For example, Strelcheck et al.^68^ report a mean dispersal of 2.1 km and a maximum of 201 km for 4,317 red snapper dart tagged at ARs off Alabama.

AT studies and networks are rapidly increasing our ability to monitor marine fish movements in a time of changing marine conditions and increased recognition of the need to integrate spatial processes into management^70–72^. AT networks have improved the spatial scale over which highly migratory fish can be tracked, but the next challenge is standardizing AT methods^10^ enough to make it possible to synthesize results across studies and thus larger spatial scales for resident species. The varying inference power of previous red snapper AT studies made that difficult in this study. Young adult red snapper commonly make small movements and are resident at sites with structure, until they are not, and there remains a need to better understand the cues, other than storms, that drive long-distance movements. Our residence time estimates of 1.4 y (50%), 1.7 y (40%) and 5.6 y for population replacement suggest lower reproductive isolation than previously thought and that heavy fishing at ARs in the GOM will affect the larger red snapper GOM stock.

## Methods

### Study site and AT sampling design

Depths in the study area ranged from 34 to 38 m. Acoustic reflectance of the seabed in an area of 65.6 km^2^ was mapped with side-scan sonar (Klein 3900, 445 kHz, with approximate swath range per channel of 100 m) to develop a fine-scale map of the distribution of bottom habitat. Bottom habitat features were ground-truthed with underwater video and included low-relief (< 0.5 m) hard bottom habitat, an AR (a sunken barge), and a reef ledge. Surveys to estimate abundance were conducted at six sites in the study area with a VideoRay Pro4 remotely operated vehicle (ROV) fitted with a forward-facing GoPro Hero 7 camera to capture 118° viewing angle. ROV transects were 3 min long and flown approximately 1 m above the seabed in the spring (May 2021 and 2022), summer (July 2020 and 2021), and winter (December 2021 and 2022).

Our array design addressed tradeoffs between optimizing location accuracy and the area and habitat types (HB, ledge, AR) that could be monitored. Limiting factors were the budget for field days to deploy/maintain the array (approximately 10 receivers per day) and receivers (*n* = 67) that could be used in the study depths: VR2AR receivers or Innovasea VR2Tx deployed in modified stands that could be recovered by ROV with a specialized arm attachment^73^. APS1 was designed with receivers deployed 600 m apart, based on detection efficiencies > 50% at a distance of 700 m in previous red snapper studies with similar tags^33,34,41^. Receivers monitoring HB and the AR were contiguous, whereas the northernmost receivers in the ledge component were 850 m away from receivers in the other components (Fig. 2A). All receivers were deployed on concrete moorings, approximately 1.5 m off the seafloor and internal receiver synchronization (sync) tags were set to 160 dB and signal transmissions ranged from 540 to 660 s, with a nominal transmission of every 10 min.

The efficacy of APS1 was impacted by multiple issues, including the COVID19 pandemic precluding deployment of the full array at the start of the study, displacement and loss of receivers due to shrimp trawling, and lower than expected detection efficiency. Although the study period was 3/27/2020 to 10/24/2022, the number and array configuration differed over this time period. Most of the receivers making up APS1 were deployed from 3/27/2020 to 4/5/2021, but only one receiver was deployed at the ledge. The remaining ledge receivers (*n* = 16) were deployed on 11/27/2020. A second APS array (APS2, *n* = 64 receivers) was deployed from 5/19/2022 to 10/24/2022. In between the APS arrays, an interim array of receivers was deployed with one receiver at each tag release site to monitor red snapper presence/absence.

Shrimp trawling caused the loss of data from 11 receivers in APS1, primarily near the ledge and AR habitats (Fig. 2A). Distances receivers were displaced was based on the positions of their sync tags. For four receivers displaced > 500 m, we retained data prior to displacement. One receiver was opportunistically displaced to the ledge, where other receivers were lost. This receiver was given a new station name after it arrived at the ledge and the data retained at its new location. Because small displacements (< 70 m, *n* = 2) resulted in receivers monitor the general deployment site, all data was retained. In February 2021, receivers considered at high risk of being lost to shrimpers were retrieved (*n* = 19) and the receiver monitoring the ledge was moved closer to the ledge to decrease the probability of it being trawled. The remaining APS1 receivers were retrieved on 4/6/2021 and 4/7/2021. An interim array was deployed with receivers at tagging sites (Fig. 2B). Multiple interviews with shrimp fishermen indicated shrimping in the study area was greatest during winter months, thus APS2 was deployed in late May/early June 2021.

The array design for APS 2 was slightly modified from APS1. APS1 had fairly low position yield, even though sync tags showed the expected detection efficiency. In an effort to maintain a similar array footprint, but increase position yield, receivers were placed 550 m apart, resulting in a larger gap (1700 m) between receivers monitoring the ledge and those monitoring HB and the AR. Because few fish were detected southwest of the ledge in APS1, this area was not monitored in APS2. Sync tag power levels were similar to implanted tags but to confirm they could be used to estimate range, we also deployed a reference tag in APS2 (same specifications as the implanted tags). In APS2, two receivers malfunctioned and an additional two receivers could not be located and were not recovered (Fig. 2C). The three habitats monitored had widely different footprints (Fig. 2D).

Slocum Electric gliders (Teledyne Webb Research) fitted with a VEMCO Mobile Transceiver^74^ were flown both within and outside of the study area to increase the spatial scale of AT tag detection (Figure S4). From 9/8/2021 to 3/7/2024, the glider flew 13 multi-day missions and was programmed to make multiple transects within and around the study area on each mission. The distance of the cumulative paths was 381 linear km, and the maximum convex hull around the paths was 11,926 km^2^. Tags detected by the glider were assumed to be live fish if there were multiple detections and observed changes in depth were consistent with expected red snapper water column use.

Mature red snapper, based on the estimated size at 50% maturity in the GOM^75^ (255 mm TL), were implanted with Innovasea acoustic tags with pressure sensors. Tags were either V16P-4x-069k-1–68 m tags (158 dB, 2,196 d battery life, interpulse delay 60–180 s with a mean of 120 s), or V13P-1x-BLU-1–0068 m tags, with 447 d battery life and the same remaining specifications as the V16P tags. All fish were captured with hook and line and implanted with acoustic tags following the surgical process described in Lowerre-Barbieri et al.^4^. Each fish was measured for total length (TL), dart tagged, and released. There was no monetary reward offered for recaptured dart tags. Releases were conducted with a descender device to return fish to depth, and video-recorded with a GoPro Hero7 camera to view potential predation events at release. None were observed. Recapture events reported by anglers to the Florida recapture hotline were collected through March 2024.

### Data analysis

To evaluate detection efficiency, we estimated the 50% detection range for our reference tag and sync tags, as well as the mean daily environmental noise at 69 kHz. All means are presented ± one standard deviation. To obtain animal relocations, we used three location estimators: presence/absence, centers of activity^12^ (COAs), and fine-scale positions for both APS arrays. Position estimates were based on the full APS1 and APS2 arrays (i.e., all array components treated as one array). COAs were calculated after spurious detections were removed (one detection in isolation within an hour) and retained if they were based on three or more receivers, with the exception of the last date of detection when emigrating red snapper often moved rapidly and had low detections. Depending on the analytical objective, COAs were calculated over 30 min or four hours (see below).

The relationship of twice the distance root mean squared (2DRMS) to horizontal position error (HPE) was used to censor positions with high error potential^41,76,77^. The 2DRMS metric relates Innovasea-provided horizontal potential error potential (HPE; unitless) to horizontal position errors (HPEm, m) from tags with known locations. We used reference sync tags that overlapped with fish positions (Fig. 3) in six array regions to estimate area-specific relationships between 2DRMS and HPEm (Figure S1, Table S1). We used the following formulation to calculate 2DRMS^78^.

$$2DRMS_{i} = 2\sqrt {\frac{1}{{n_{i} }}\sum\nolimits_{{j = 1}}^{n} {({{\varDelta}}\:x_{{i,j}}^{2} + {{\varDelta}}\:y_{{i,j}}^{2} )} } ,$$

where $\:\varDelta\:{x}_{i,j}$ and $\:\varDelta\:{y}_{i,j}$ are the error in the east-west and north-south components of the *j*th observation in HPE bin *i*, and *n* is the number of observations per HPE bin of increment 1. To avoid undue influence of outliers, the upper 5% of HPE values were censored prior to 2DRMS calculation^41^. 2DRMS was regressed against mean HPE per bin, including an ‘array region’ interaction term which was defined at various spatial scales within array in alternative model formulations. Model comparison was used to determine the best fit, and thus the appropriate number of 2DRMS relationships and HPE filters needed per array.

*Fate assignment*. Fates were assigned based on recapture histories, glider detections outside the array, and horizontal and vertical movement signatures. Movement signatures were visualized using an R Shiny application that made it possible to view for each fish its COA and depth locations over the study based on receiver and glider detections. The application also allowed the user to “zoom in” over shorter time scales to better assess fine-scale movements (Figure S5). This was especially helpful for the last several days of detections. Confirmed movements of red snapper (later recaptured) were used as baseline red snapper horizontal and vertical behavior. We assumed a three-day recovery period from the stress of capture, tagging, and release (CTR) and fates occurring in this time period were assigned a CTR prefix.

Movement signatures varied in their interpretability. Stationary tags were most easily flagged based on minimal change in depth (standard deviations of < 0.25 m) and were assigned as mortality if they were not preceded by a predation movement signature. Predation movement signatures mimicked behaviors common to sharks in terms of water column use and/or large horizontal space use^46^. Predation was assigned if the predation signature was observed prior to the tag becoming stationary or leaving the array. However, these signatures were only apparent if a fish was predated in the central part of the HB array component. Capture removal was based on recapture events or detections that stopped abruptly within the middle of the HB component of an APS array. Emigration was assigned on the date a tag was no longer detected for the remainder of the study if any of the following occurred: (1) ‘typical’ movement signatures within the a red snapper’s range followed by movements towards the edge of the array were observed; (2) the fish was confirmed as having emigrated (i.e., recaptured or detected alive by the glider outside of the array); or, (3) multiple tags left the array within a 48 h time period and at least one was confirmed as having emigrated. An unknown fate was assigned to tags that stopped being detected but the cause could not be determined. Fish with unknown fates could have emigrated, been captured, or predated. Final detection dates of fish with emigrated and unknown fates were assessed for seasonal patterns. Fish that exhibited red snapper movement signatures throughout the study period were assigned as surviving. Fates were assigned independently to each fish by three scientists based on movement patterns and recapture histories. Percent agreement was calculated. If fates differed amongst scientists, a final consensus fate was assigned based on group review of the indicators.

*Site fidelity.* Observed residence times are affected by tagging dates, array deployment dates, and the spatial scale of interest. Here we define potential residence time as the maximum tracking duration possible if all fish survived and remained within the array. This was calculated as the last day of the study minus each fish’s tagging date. Traditional residence indices (RIs) were calculated for each fish as the number of detection days (DD) divided by the total detection period (i.e., the number of days from the first to last detection day)^79^. Given the differences in receiver coverage over this study, we use daily detection gaps to identify when receiver coverage was not sufficient to monitor fish movements within the study area (i.e., > 1 daily detection gap). Habitat-specific observed residence times were calculated as the last date the fish was detected (as a red snapper) minus its tagging date for fish that did not change habitat, had no more than one daily detection gap, and a fate of survival, emigration, or unknown. These were compared to habitat-specific potential residence times. The number of fish tracked after the tagging recovery period (3 d) is reported.

Annual site fidelity refers to the proportion of fish remaining in the study area after a year. Detection data of all tracked fish was used, regardless of array, if the fish had no more than one daily detection gap. Two methods were used to estimate annual site fidelity to the study area: (1) event history analysis where emigration is the event, and (2) a binomial daily emigration probability (DEP) generalized linear model (GLM) that included time-varying covariates. For event history analysis, we used both empirical and parametric approaches. For empirical estimators, we used the Kaplan-Meier(K-M) product limit algorithm^80^ for right-censored data as implemented in the R package ‘prodlim’^81^. For parametric estimators, a Weibull model was fitted using the ‘survival’ R package^82^. All event histories started on day zero, the tagging date. All fish detected after the three-day tag recovery period and with no more than one daily detection gap were included. Fates other than emigration are right-censored and removed from the analysis on the date the fate occurred. To evaluate the impact of fate assignment on results, we repeated the analysis setting ‘unknown’ fates to ‘emigration’.

For the DEP model, we evaluated three candidate environmental variables that may cue large-scale movements associated with storm events and retained the best predictor (Figure S6). We obtained atmospheric pressure data from the NOAA St. Petersburg (Florida, USA) weather station. Bottom water temperature and receiver tilt (used as an indicator of current) data came from the receivers’ sensors. The best predictor was atmospheric pressure (Table S4). Median daily atmospheric pressure was calculated for all study dates and dates were assigned as regular or low-pressure dates based on days when pressures were < 0.5th percentile (1007.96 millibar) of daily median values over the study period. Data were summarized as the total number of fish detected on a calendar date and total number of fish emigrated on that calendar date. Atmospheric pressure was included as a binary covariate signifying regular and low atmospheric pressure dates. The model had the form:

$$E_{i} \sim Binom(n_{i} p_{i} )$$

$$\:logit\left({p}_{i}\right)=\:\beta\:{P}_{i}$$

where *E* is the number of emigrations on day *i*,* n* is the number of fish observed in the array that day, and *p* is the probability of emigration. Atmospheric pressure is denoted as *P*. As with event history analysis, fate uncertainty was evaluated. The fitted models were used to calculate the probability of emigration on regular and lower pressure days (*P*_*r*_ and *P*_*l*_, respectively). From that, expected site fidelity after t days can be obtained for a given number of lower pressure days (*D*_*l*_ ) as:

$$\:S\left(t\right)={(1-{p}_{r})}^{\left(t-{D}_{L}\right)}*{(1-{p}_{l})}^{{D}_{L}}$$

### Space use and movement patterns

We used several measures to evaluate red snapper space use. Utility distributions (UD) were estimated at the 95% contour at the daily and tracking duration temporal scales. Daily UDs (UD_d_) were calculated as Brownian Bridges for position estimates occurring within 1 h of each other with the R package adehabitatHR^83^ using a grid size of 10 × 10 m. Location error size was based on our assigned appropriate threshold of position error. Daily UDs were weighted averages of UDs calculated for consecutive positions occurring within a 24-hour period, with no more than 60 min between each position, where each UD was weighted based on the time interval it encompassed. To assess space use at the tracking duration scale, a single 95% contour UD for each fish was calculated based on a weighted average of the daily contour UDs and areas(UD_td_). To avoid undue influence of low-data fish, UDs were only calculated for fish that had a minimum of 33 d for which UDs could be estimated (the 90% quantile), and for which the mean number of daily positions was at least 18 (again, the 90% quantile). High density use sites or “centroids” were identified within individual UD_td_ based on 90% contours and a minimum size of 100 m^2^. To evaluate if fish shifted centroid use over the tracking duration, the number of daily positions in each centroid was calculated and a shift in space use was defined as the use of a new centroid (s) that did not overlap temporally with prior centroid use. Because space use often increases with tracking duration^35^, and tracking durations varied between habitats, we assessed if there was a relationship between UD_td_ and the number of days a fish was tracked before evaluating the UD_td_ and patch size relationship. Variables were tested for normality using the Shapiro-Wilk test. UD_td_ were not distributed normally (*p* < 0.0001) and thus the nonparametric Spearman’s rank correlation coefficient was used to evaluate relationships between UD_td_ and days tracked, as well as to see if space use increased with patch size.

To better understand less common movement patterns, we evaluated distances traveled and their associated movement rates (m/sec), and the occurrence of long-distance movements (LDMs). LDMs were defined as the maximum distance between any two locations within 48 h exceeding 2000 m, a definition that considers both a distance and temporal threshold to distinguish it from more gradual position shifts over large distances. Movement rate was calculated as observed distances between each successive pair of positions divided by the time between positions. A corrected movement rate was also calculated, assuming the worst possible position error (i.e., decreasing the distance between positions by two times the accepted position error) to help validate movement rates in this study > 0.75 m/sec, the maximum reported in Bohaboy et al.^46^ To compare tradeoffs between location accuracy and the ability to identify LDMs, both position data and COA data were used. The time step for the COAs was increased to 4 h. This was needed because of the gap in receiver coverage between the ledge and HB. Without a longer time step fish appeared to be moving from the ledge to HB or back when in fact they were located between these areas and minor shifts in location resulted in detection on the edge of one or the other habitats (Figure S7). The total number of LDMs and the number of LDMs per fish were estimated and connectivity between array components assessed.

*Dispersal.* Maximum individual dispersal over the course of the study was calculated as the maximum distance between any two estimated locations, where estimated locations included 4 h COAs as well as recapture locations and glider detections of live red snapper outside the array. Only fish with a tracking period (from tagging to final detection or recapture) of at least 100 days were included. The R package ‘fitdistrplus’^84^ was used to fit probability distributions to the observed maximum dispersal distances from which to estimate the probability of moving a certain distance over the course of the study period. Eight of the emigrated fish were not subsequently relocated by recapture or the glider, and the maximum distances for those fish (from acoustic receiver detections within the array) were treated as right-censored since we know the fish moved outside of the array. Several candidate distributions were fit and model comparison was used to determine the best-fitting one.

*Comparing red snapper movement traits across AT studies.* To assess if movement trait results could be compared between this study and previous studies we reviewed literature on red snapper AT studies in the GOM since 2011 for site fidelity and fate results, as well as AT design components that would affect them. The design components assessed were: number of tags deployed versus number of fish tracked after the recovery period, fish size, number of arrays and receivers deployed, array deployment days, maximum receiver depth, and estimated detection probabilities.

## Electronic supplementary material

Below is the link to the electronic supplementary material.

Supplementary Material 1

## Data Availability

Due to ethical concerns about making the precise locations of red snapper available to the public, data are available upon reasonable request to the corresponding author (Slowerrebarbieri@ufl.edu) in the manuscript.
