# Supplementary material for: Movement traits important to conservation and fisheries management: an example with red snapper
Source: Sci Rep. 2025 Feb 7;15:4614. doi: 10.1038/s41598-025-86892-1 (PMC11805965; doi:10.1038/s41598-025-86892-1)

**Supplementary material**

*Daily Emigration Probability Modeling*

Tilt and water temperature data were retrieved from the receivers. Tilt data was considered a proxy for bottom current strength. To calculate mean daily tilt, receiver-specific tilt time-series were first inspected for problematic sensor data and those receivers removed. To standardize tilt due to varying rugosity and receiver type (Tx vs AR) each receiver’s tilt value was subtracted from its minimum tilt, and then daily averages across receivers were calculated. Sudden changes in bottom temperatures were identified by evaluating the maximum difference between three-day rolling mean daily temperature values. Each of the three environmental indicator variables were converted into binary predictors of conditions that would be expected to trigger long distance movements using the 0.5^th^ (pressure) and 99.5^th^ (tilt and bottom temperature) percentiles of the data daily values over the study period as the threshold: for atmospheric pressure a median value less than 1007.96, for tilt a mean value of 6.57 or more, and for temperature differences a value of 1.64 degree Celsius or more (Figure S6). The number of long-distance movements were enumerated by Date, array component, and the three binary environmental data predictor variables. They were modeled as having a binomial distribution with the number of trials (*n*) being equal to the number of fish with detections for a given Date. The most parsimonious model included only atmospheric pressure (P) as a predictor (Table S4):

$${LDM}_{i}\sim Binom\left( n_{i},p_{i} \right)$$

$$logit\left( p_{i} \right)=\beta P_{i}$$

*Sensitivity of Brownian Bridge UD Estimates to Position Error*

We evaluated Brownian Bridge movement model sensitivity to position error using a quick simulation. For a movement track simulated over the period of ~4 months over an area of ~7900 m^2^, the Brownian Bridge UD estimates varied depending on the true location error and the location error assumed for estimation. The smallest tracking duration UD estimate of 8078 m^2^ was obtained for a true and assumed location error of 3 m. The largest HR estimate of 24,679 m^2^ was obtained for a true mean location error of 20 m and an assumed error of 30 m (Table S3). For a true and assumed error of 20 m, the estimate was 19,691 m^2^

*Supplemental Tables and Figures*

Table S1. Model selection table for determining the number of 2DRMS relationships to use for censoring fish APS data based on subdividing each APS into separate regions (see Fig2 in the main text). The best fitting model estimated a single relationship for APS1 and separate relationships for each area in APS2. The chosen model was one where, in addition, the beacon tag in APS2 was combined with the reference tags in the region where it was located (the SW). This model was comparable to the best-fitting model (delta AIC = 1.71).

| model | df | logLik | AICc | DAICc | weight |
| --- | --- | --- | --- | --- | --- |
| APS1: single, APS2: separate | 17 | -426.8 | 892.5 | 0.00 | 0.698 |
| *APS1: single, APS2: separate but beacon grouped with SW* | *15* | *-430.2* | *894.2* | *1.71* | *0.296* |
| Separate relationships for each array area | 23 | -423.4 | 902.1 | 9.55 | 0.006 |
| Separate relationships for WR and CS by APS, beacon grouped with SW | 13 | -532.8 | 1094.5 | 201.93 | 0.000 |
| One relationship per APS | 5 | -549.9 | 1110.2 | 217.65 | 0.000 |
| APS2: single, APS1: separate | 11 | -549.3 | 1122.6 | 230.05 | 0.000 |
| single relationship for both APS combined | 3 | -592.1 | 1190.5 | 297.94 | 0.000 |

Table S2. Information on acoustically tagged fish, including ID, tagging location, fish size at tagging, fate, tagging date, fate date, potential and realized tracking duration, days detected, and residence index (RI).

| **Tag Number** | **Tagging Location** | **TL** | **Tagging Date** | **Fate Date** | **Potential Tracking Duration** | **Realized Tracking Duration** | **Days Detected** | **RI** | **Fate** |
| --- | --- | --- | --- | --- | --- | --- | --- | --- | --- |
| 1 | HB | 430 | 3/27/2020 | 3/27/2020 |  |  |  |  | ctr_predation |
| 2 | HB | 515 | 3/27/2020 | 9/7/2020 | 942 | 165 | 165 | 1.00 | capture_removal |
| 3 | HB | 459 | 3/27/2020 | 9/7/2020 | 942 | 164 | 164 | 1.00 | capture_removal |
| 4 | HB | 546 | 3/27/2020 | 11/29/2020 | 942 | 248 | 248 | 1.00 | mortality |
| 5 | HB | 572 | 3/27/2020 | 3/27/2020 |  |  |  |  | ctr_predation |
| 6 | HB | 498 | 3/27/2020 | 1/15/2021 | 942 | 295 | 295 | 1.00 | emigrated |
| 7 | HB | 572 | 3/27/2020 | 10/24/2022 | 942 | 942 | 941 | 1.00 | survived |
| 8 | HB | 537 | 3/27/2020 | 10/29/2021 | 942 | 582 | 534 | 0.92 | emigrated |
| 9 | HB | 466 | 3/27/2020 | 7/15/2021 | 942 | 476 | 476 | 1.00 | predated |
| 10 | HB | 521 | 3/27/2020 | 3/27/2020 |  |  |  |  | ctr_mortality |
| 11 | HB | 520 | 3/27/2020 | 3/27/2020 |  |  |  |  | ctr_mortality |
| 12 | HB | 525 | 3/27/2020 | 6/3/2021 | 942 | 434 | 434 | 1.00 | unknown |
| 13 | Ledge | 577 | 12/22/2020 | 5/10/2021 | 672 | 140 | 140 | 1.00 | unknown |
| 14 | Ledge | 504 | 12/22/2020 | 5/28/2021 | 672 | 158 | 158 | 1.00 | unknown |
| 15 | Ledge | 495 | 12/22/2020 | 6/17/2022 | 672 | 543 | 469 | 0.86 | capture_removal |
| 16 | Ledge | 629 | 12/22/2020 | 3/8/2021 | 672 | 77 | 77 | 1.00 | mortality |
| 17 | Ledge | 764 | 12/22/2020 | 2/28/2021 | 672 | 69 | 69 | 1.00 | emigrated |
| 18 | Ledge | 503 | 12/22/2020 | 10/24/2022 | 672 | 672 | 589 | 0.88 | survived |
| 19 | Ledge | 481 | 12/22/2020 | 10/29/2021 | 672 | 312 | 286 | 0.92 | emigrated |
| 20 | Ledge | 473 | 12/22/2020 | 7/13/2022 | 672 | 569 | 444 | 0.78 | emigrated |
| 21 | Artificial | 470 | 1/13/2021 | 5/31/2021 | 650 | 139 | 139 | 1.00 | mortality |
| 22 | Artificial | 478 | 1/13/2021 | 1/13/2021 |  |  |  |  | ctr_predation |
| 23 | Artificial | 457 | 1/13/2021 | 6/6/2021 | 650 | 145 | 145 | 1.00 | mortality |
| 24 | HB | 457 | 1/13/2021 | 10/29/2021 | 650 | 290 | 290 | 1.00 | emigrated |
| 25 | HB | 477 | 1/13/2021 | 4/26/2021 | 650 | 104 | 104 | 1.00 | mortality |
| 26 | HB | 501 | 1/13/2021 | 7/28/2021 | 650 | 197 | 197 | 1.00 | unknown |
| 27 | HB | 513 | 1/13/2021 | 10/24/2022 | 650 | 650 | 650 | 1.00 | survived |
| 28 | HB | 489 | 1/13/2021 | 10/29/2021 | 650 | 290 | 290 | 1.00 | emigrated |
| 29 | HB | 610 | 1/13/2021 | 5/3/2021 | 650 | 111 | 111 | 1.00 | unknown |
| 30 | HB | 525 | 1/13/2021 | 7/17/2021 | 650 | 186 | 186 | 1.00 | capture_removal |
| 31 | HB | 530 | 1/13/2021 | 7/14/2021 | 650 | 183 | 183 | 1.00 | capture_removal |
| 32 | HB | 476 | 1/13/2021 | 8/12/2022 | 650 | 577 | 577 | 1.00 | predated |
| 33 | Ledge | 507 | 12/3/2021 | 10/24/2022 | 326 | 326 | 318 | 0.98 | survived |
| 34 | Ledge | 564 | 12/3/2021 | 10/24/2022 | 326 | 326 | 326 | 1.00 | survived |
| 35 | Ledge | 574 | 12/3/2021 | 5/29/2022 | 326 | 178 | 178 | 1.00 | unknown |
| 36 | Ledge | 500 | 12/3/2021 | 10/24/2022 | 326 | 326 | 326 | 1.00 | survived |
| 37 | HB | 553 | 12/3/2021 | 9/28/2022 | 326 | 300 | 300 | 1.00 | emigrated |
| 38 | Artificial | 497 | 12/3/2021 | 6/20/2022 | 326 | 200 | 200 | 1.00 | capture_removal |
| 39 | Artificial | 488 | 12/3/2021 | 12/3/2021 |  |  |  |  | tag_fail |
| 40 | Artificial | 543 | 12/3/2021 | 12/3/2021 |  |  |  |  | tag_fail |
| 41 | HB | 545 | 12/7/2021 | 10/24/2022 | 322 | 322 | 322 | 1.00 | survived |
| 42 | HB | 494 | 12/7/2021 | 10/24/2022 | 322 | 322 | 322 | 1.00 | survived |
| 43 | HB | 494 | 12/7/2021 | 10/24/2022 | 322 | 322 | 322 | 1.00 | survived |
| 44 | HB | 515 | 12/7/2021 | 6/20/2022 | 322 | 196 | 196 | 1.00 | emigrated |
| 45 | HB | 530 | 12/7/2021 | 10/25/2022 | 322 | 322 | 192 | 0.60 | survived |
| 46 | Artificial | 733 | 1/20/2022 | 1/20/2022 |  |  |  |  | ctr_predation |
| 47 | HB | 504 | 1/20/2022 | 5/15/2022 | 278 | 116 | 116 | 1.00 | unknown |
| 48 | HB | 602 | 1/20/2022 | 10/25/2022 | 278 | 278 | 278 | 1.00 | survived |
| 49 | HB | 574 | 1/20/2022 | 1/20/2022 |  |  |  |  | ctr_predation |
| 50 | HB | 562 | 1/20/2022 | 10/24/2022 | 278 | 278 | 278 | 1.00 | survived |
| 51 | Artificial | 445 | 1/20/2022 | 10/25/2022 | 278 | 278 | 278 | 1.00 | survived |
| 52 | Artificial | 414 | 1/20/2022 | 6/13/2022 | 278 | 145 | 145 | 1.00 | predated |
| 53 | HB | 443 | 1/20/2022 | 10/24/2022 | 278 | 278 | 278 | 1.00 | survived |
| 54 | HB | 431 | 1/20/2022 | 6/22/2022 | 278 | 154 | 154 | 1.00 | emigrated |
| 55 | HB | 435 | 1/20/2022 | 9/12/2022 | 278 | 154 | 150 | 0.97 | emigrated |
| 56 | HB | 397 |  |  |  |  |  |  | not detected |
| 57 | HB | 527 | 1/20/2022 | 10/24/2022 | 278 | 278 | 278 | 1.00 | survived |
| 58 | HB | 427 |  |  |  |  |  |  | not detected |
| 59 | HB | 388 | 1/20/2022 | 10/25/2022 | 278 | 278 | 161 | 0.58 | survived |
| 60 | HB | 656 | 1/20/2022 | 10/25/2022 | 278 | 278 | 162 | 0.58 | survived |
| 61 | HB | 404 | 1/20/2022 | 2/21/2022 | 278 | 33 | 33 | 1.00 | unknown |
| 62 | HB | 463 | 1/20/2022 | 1/20/2022 |  |  |  |  | ctr_mortality |
| 63 | HB | 436 | 6/7/2022 | 10/25/2022 | 140 | 140 | 140 | 1.00 | survived |
| 64 | HB | 425 | 6/7/2022 | 6/7/2022 |  |  |  |  | ctr_predation |
| 65 | HB | 463 | 6/7/2022 | 10/24/2022 | 140 | 140 | 140 | 1.00 | survived |
| 66 | Artificial | 443 | 6/7/2022 | 10/25/2022 | 140 | 140 | 140 | 1.00 | survived |
| 67 | Artificial | 473 | 6/7/2022 | 10/25/2022 | 140 | 140 | 140 | 1.00 | survived |
| 68 | Ledge | 504 | 6/7/2022 | 9/29/2022 | 140 | 115 | 115 | 1.00 | emigrated |
| 69 | HB | 433 | 6/7/2022 | 10/25/2022 | 140 | 140 | 140 | 1.00 | survived |
| 70 | HB | 435 | 6/7/2022 | 10/24/2022 | 140 | 140 | 140 | 1.00 | survived |
| 71 | HB | 438 | 1/20/2022 | 10/24/2022 | 278 | 278 | 278 | 1.00 | survived |
| 72 | HB | 410 | 1/20/2022 | 10/24/2022 | 278 | 278 | 278 | 1.00 | survived |
| 73 | HB | 395 | 1/20/2022 | 6/15/2022 | 278 | 147 | 147 | 1.00 | emigrated |
| 74 | HB | 416 | 6/7/2022 | 10/24/2022 | 140 | 140 | 140 | 1.00 | survived |
| 75 | Artificial | 429 | 6/7/2022 | 10/25/2022 | 140 | 140 | 140 | 1.00 | survived |
| 76 | HB | 529 | 6/7/2022 | 10/24/2022 | 140 | 140 | 139 | 0.99 | survived |
| 77 | Artificial | 522 | 6/7/2022 | 6/23/2022 | 140 | 17 | 17 | 1.00 | unknown |
| 78 | Ledge | 583 | 6/7/2022 | 6/13/2022 | 140 | 7 | 7 | 1.00 | unknown |
| 79 | HB | 613 | 6/7/2022 | 6/7/2022 |  |  |  |  | ctr_predation |
| 80 | Ledge | 519 | 6/7/2022 | 10/24/2022 | 140 | 140 | 140 | 1.00 | survived |
| 81 | Artificial | 536 | 6/7/2022 | 9/28/2022 | 140 | 114 | 114 | 1.00 | emigrated |
| 82 | Artificial | 611 | 6/7/2022 | 9/28/2022 | 140 | 114 | 114 | 1.00 | emigrated |
| 83 | HB | 507 | 6/7/2022 | 10/25/2022 | 140 | 140 | 140 | 1.00 | survived |
| 84 | HB | 516 | 6/7/2022 | 10/24/2022 | 140 | 140 | 140 | 1.00 | survived |
| 85 | HB | 545 | 6/7/2022 | 10/24/2022 | 140 | 140 | 140 | 1.00 | survived |

Table S3. Brownian Bridge Kernel UD (BBUD) space use estimates for a simulated set of points over a circular area of roughly ~7850 m^2^, showing the sensitivity of BBUD estimates to true and assumed horizontal position error. Observed points were generated from true points by adding the indicated horizontal position error as draws from a normal distribution. The assumed error is the amount of error specified in the Kernel UD calculation.

| True Error (m) | Assumed Error (m) | Estimated Space use (m^2^) |
| --- | --- | --- |
| 3 | 3 | 8,078 |
| 10 | 10 | 11,237 |
| 20 | 20 | 19,691 |
| 10 | 3 | 10,673 |
| 20 | 10 | 15,518 |
| 30 | 20 | 21,612 |
| 3 | 10 | 9,600 |
| 10 | 20 | 15,125 |
| 20 | 30 | 24,679 |

Table S4. Model comparison table for testing different binary predictors of daily emigration probability. Elpd = expected log predictive density. AtmP = Atmospheric Pressure, BT = bottom temperature, Ti = Tilt. The BT only models had convergence issues.

| **Model** | **elpd_diff** | **se_diff** | **weight** |
| --- | --- | --- | --- |
| AtmP | 0.000 | 0.000 | 0.590 |
| AtmP + BT | -0.365 | 0.292 | 0.410 |
| AtmP + BT + Ti | -6.116 | 4.048 | 0.000 |
| AtmP + Ti | -6.304 | 4.275 | 0.000 |
| Ti | -17.613 | 18.806 | 0.000 |
| Intercept only | -28.497 | 20.358 | 0.000 |

Figure S1. Estimated 2DRMS-to-HPE relationships (black) used to censor high HPE values from the APS data. The 2DRMS values used to fit the relationships are shown as black circles and the data from which the 2DRMS values were calculated are shown as blue dots up until HPE 50. The upper 95^th^ quantile of HPE values for each array area was excluded from 2DRMS calculations. The 1:1 relationship is indicated by the dashed line in each plot, and the horizontal red line represents the 20m horizontal position error level from which the specific HPE threshold for data censoring was determine for each of the indicated APS components. A single relationship was estimated for APS1 and area-specific relationships were determined for APS2.


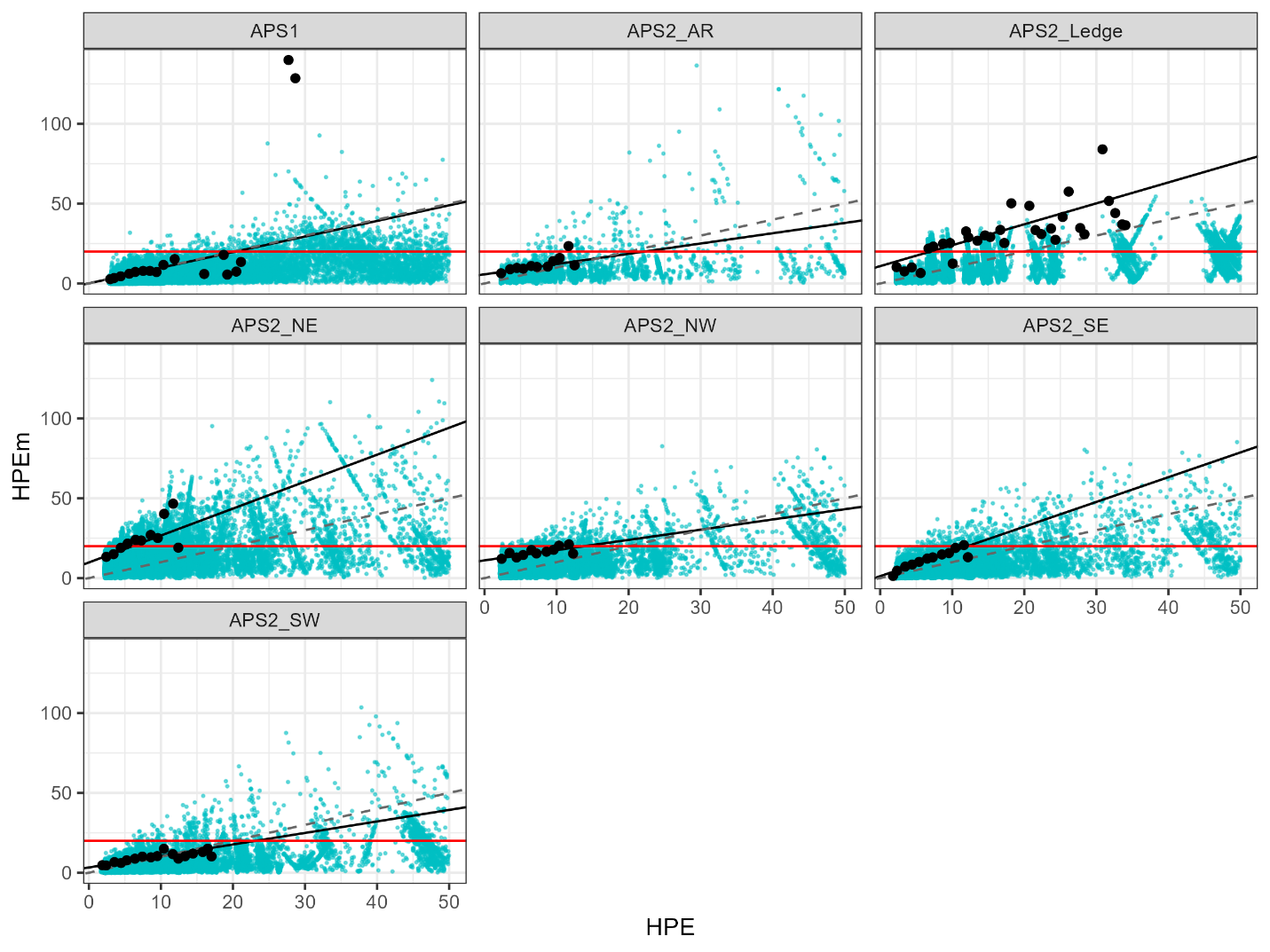


Figure S2. Estimated positions based on a) 30 min COAs, b) 4 hr COAs, and c) APS. Censored positions are shown in red. No positions were censored for COAs, based on the requirement to have at least 3 stations with detections within each time bin. For APS, positions were censored based on the 2DRMS-to-HPE relationship and a 20m desired error level. The true position of the reference tag is shown in green. Receivers (shown as grey circles) were located approximately 550 m apart.


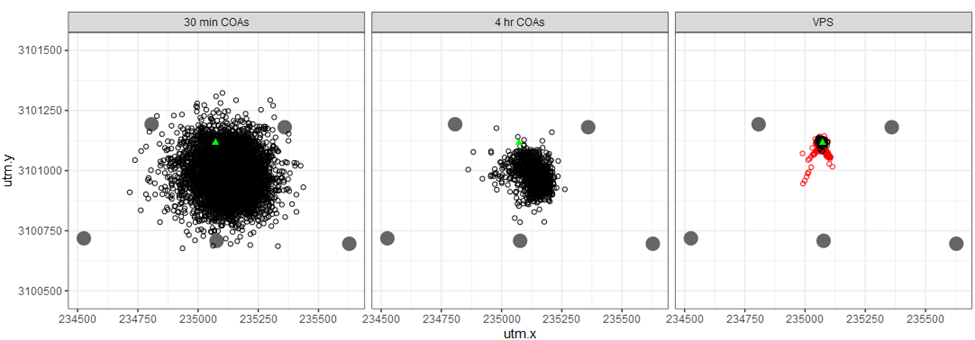


Figure 3. Dispersal modeling results. The top panel shows the fits of the various tested distributions (empirical against fitted cumulative distribution functions (CDFs), empirical against theoretical quantiles, and empirical against theoretical probabilities), with the Burr distribution being the best fit (D AIC = 24). The bottom panel shows the predicted probability of moving at least a certain distance (left plot) and the predicted probability and observed counts of moving exactly a certain distance (right plot), b ased on Burr distribution predictions.


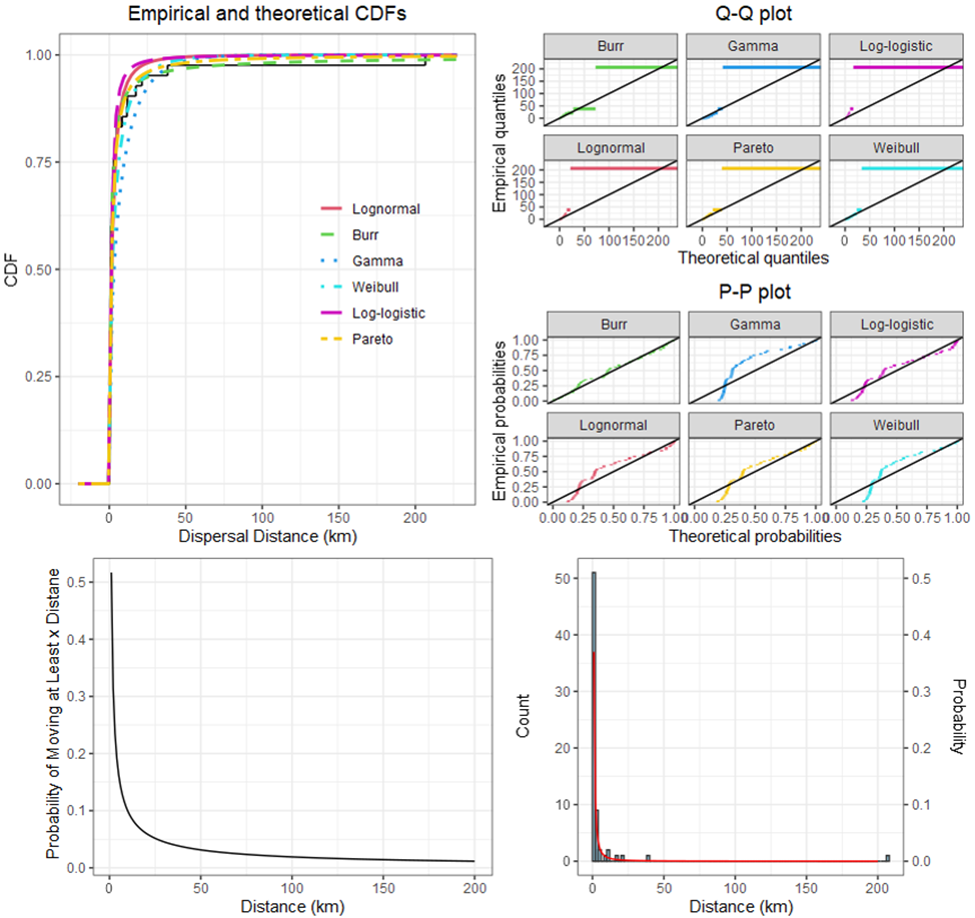


Figure S4 Pathways of the slocum glider within and near the study site.


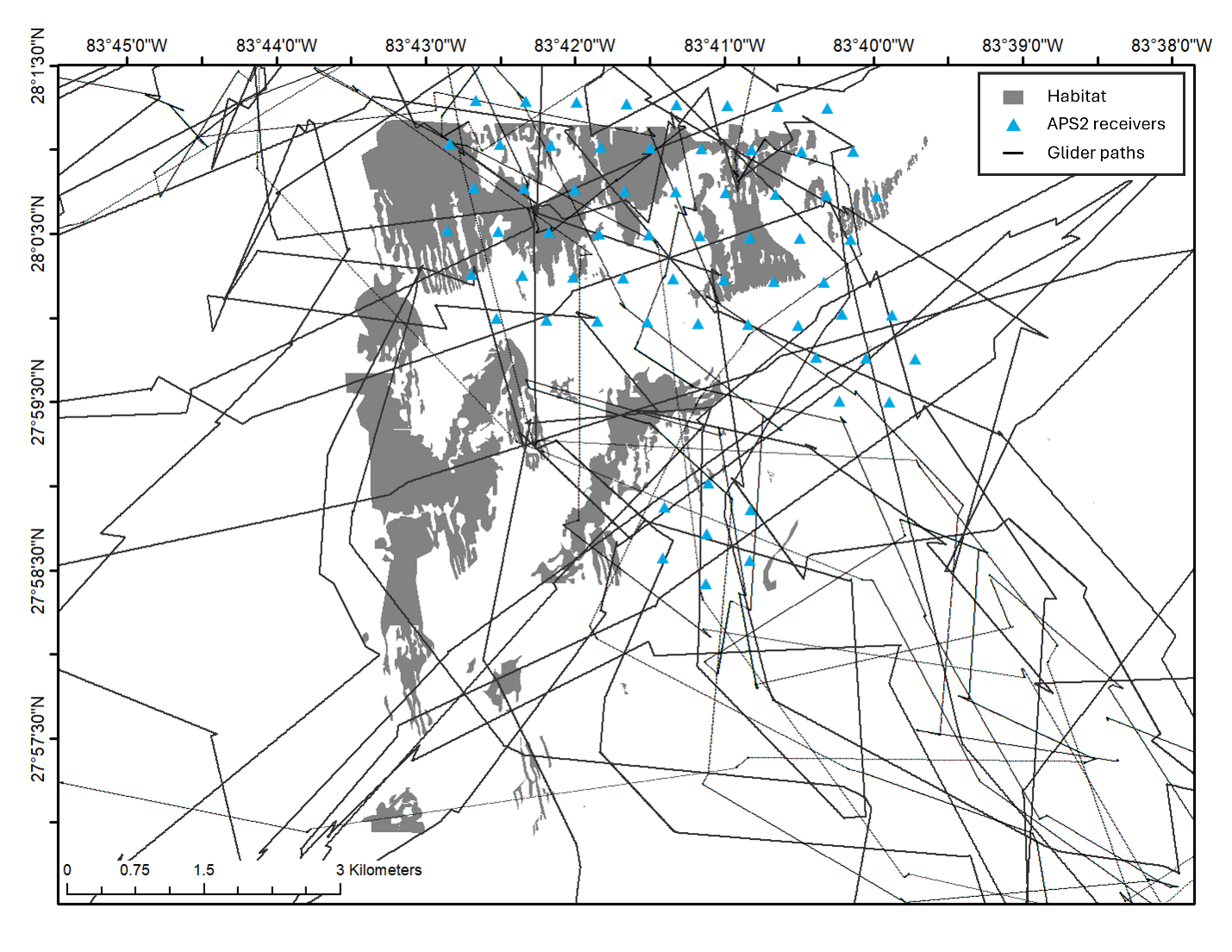


Figure S5. Examples of different fate assignments using the shiny application. Top image is an example of emigration (tag 5807) that includes use of the glider data (right panel), middle is an example of CTR predation (tag 4900,) and last is an example of mortality, where a fish dies and its tag is consistently detected on the bottom (tag 4911).


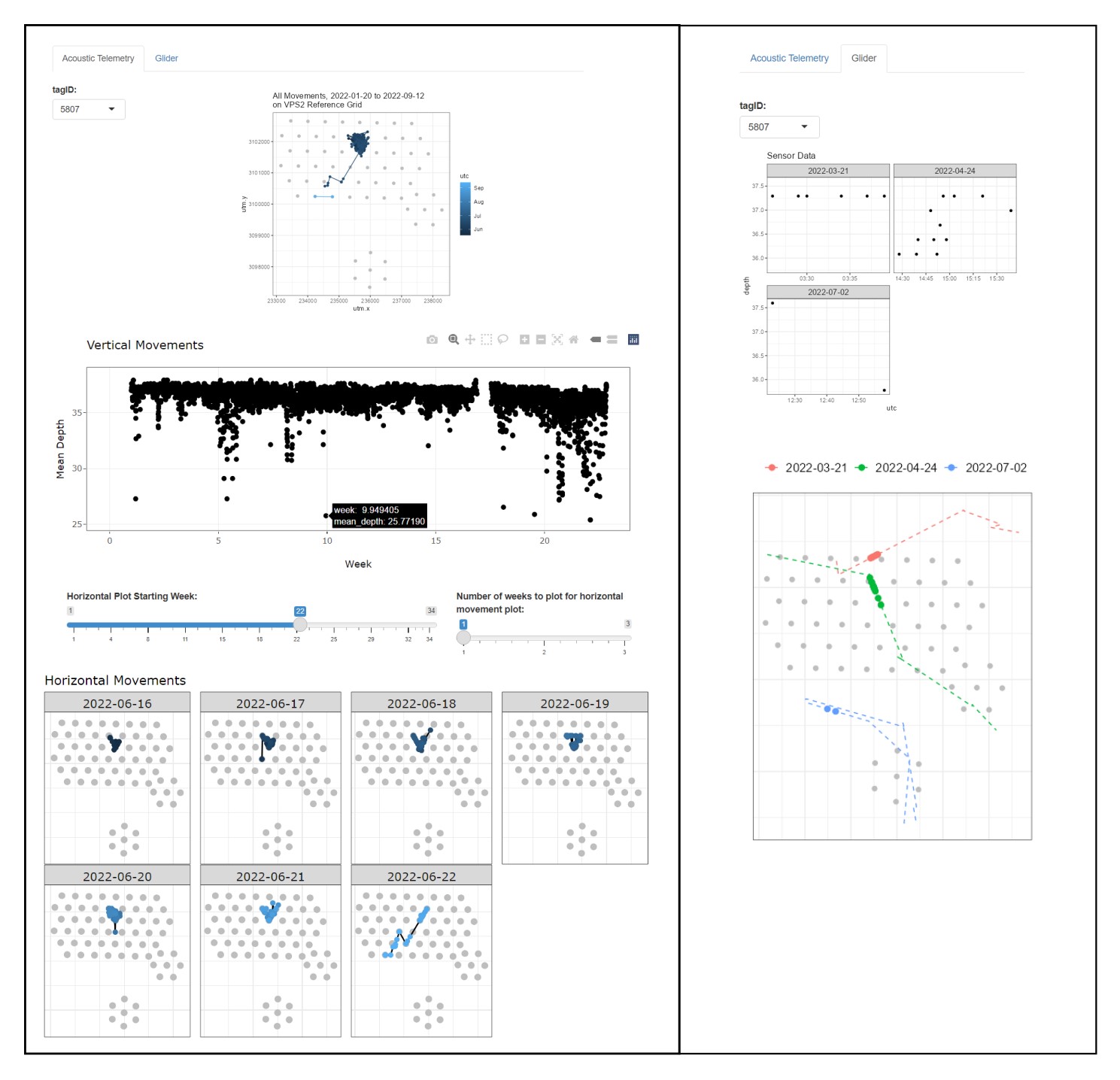


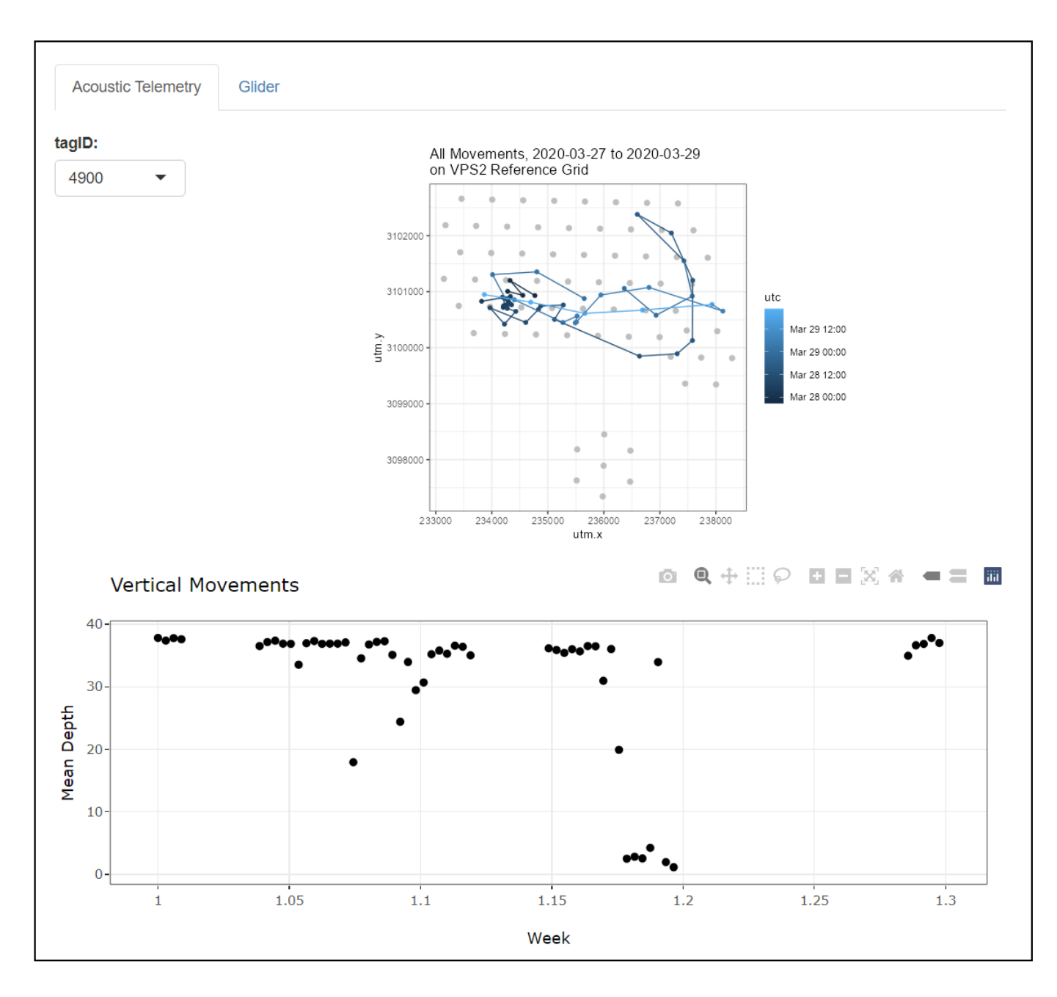

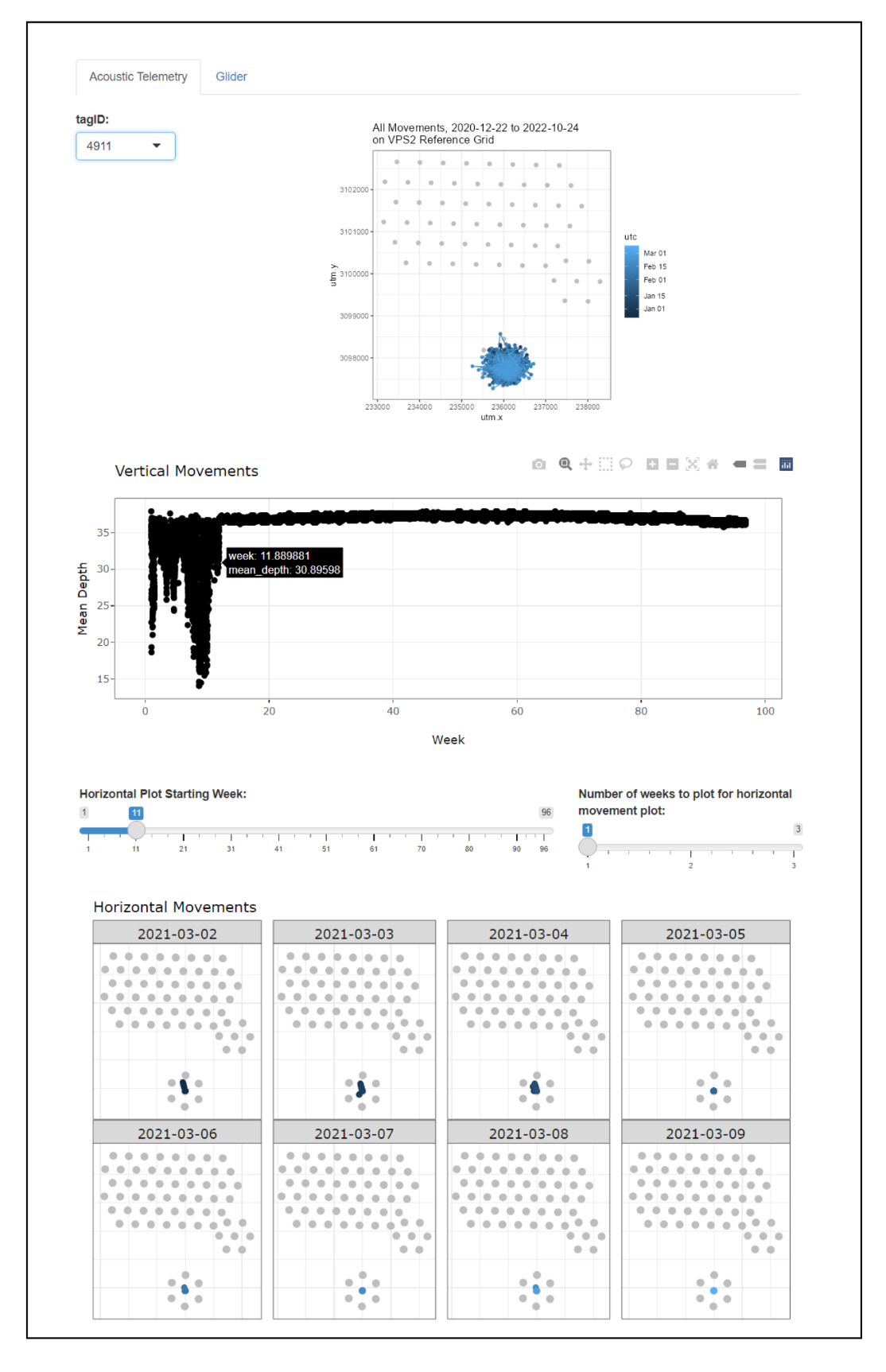


Figure S6. Time series for environmental covariates tested in the daily emigration probability model. The blue lines are the thresholds for converting each variable to binary categorical variables. The red lines are the dates of synchronized emigration.


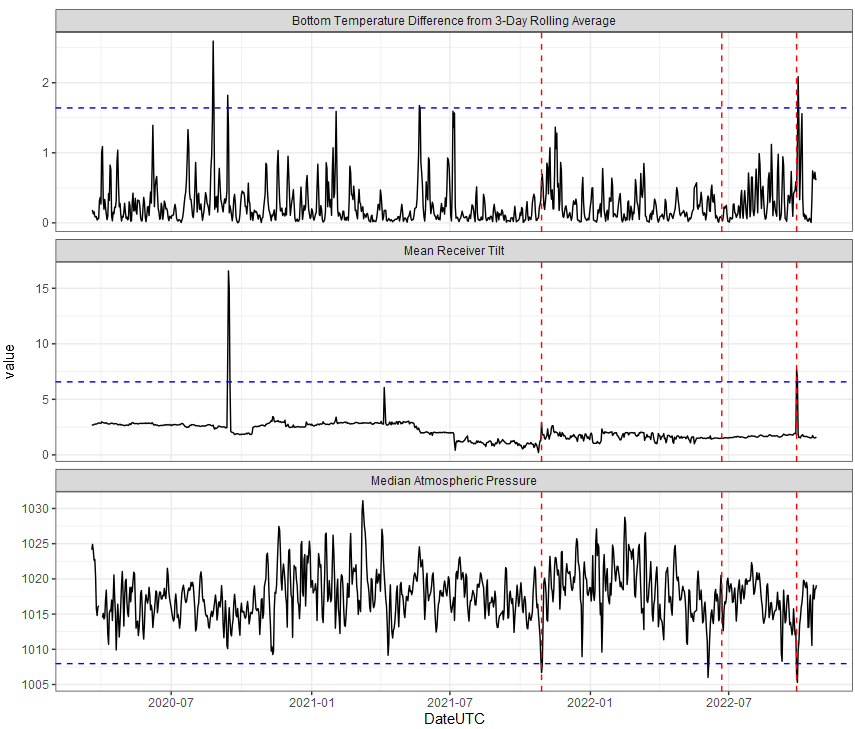


Figure S7. COA results for different COA bin periods (in minutes) for a single tag over a 24 hour period showing how variable detection ranges and potentially true fish position in between the ledge and HB/artificial reef components can give the appearance of movement between array components at small bin intervals.


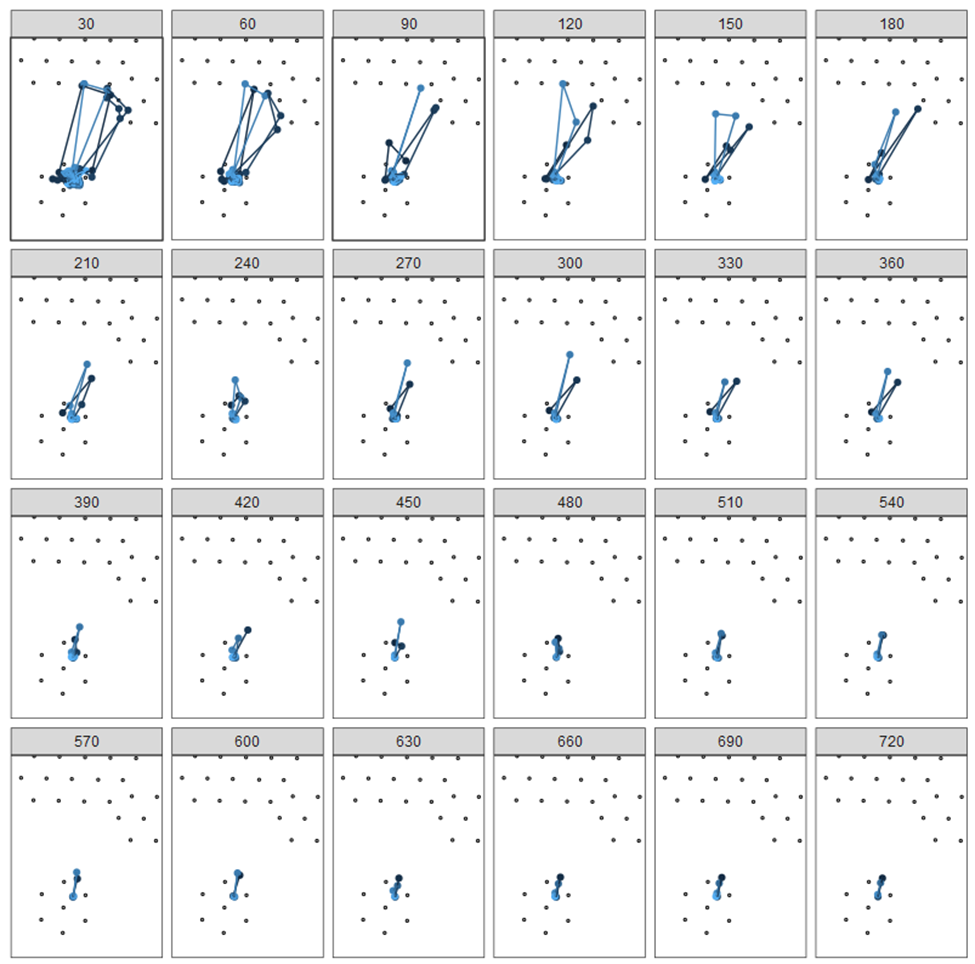

Supplement: Supplementary file 1 — Supplementary Material 1 [file 41598_2025_86892_MOESM1_ESM.docx]
